# Heterologous Expression Unexpectedly Activates the Host Cryptic Genes in *Aspergillus nidulans* and Enables the Discovery of Novel Natural Products

**DOI:** 10.3390/jof12060401

**Published:** 2026-06-01

**Authors:** Cong Liu, Yinan Hao, Siyuan Qi, Jian Bai

**Affiliations:** State Key Laboratory of Bioactive Substance and Function of Natural Medicines, Institute of Materia Medica, Chinese Academy of Medical Sciences & Peking Union Medical College, Beijing 100050, China; liucong@imm.ac.cn (C.L.); haoyinan@imm.ac.cn (Y.H.); qisiyuan@imm.ac.cn (S.Q.)

**Keywords:** *Aspergillus nidulans*, metabolic perturbation, silent gene activation, diketopiperazine–isoindolinone hybrid alkaloids, asperfuranic acid, antioxidant activity

## Abstract

*Aspergillus nidulans*, a model filamentous fungus endowed with well-established genetic tools and a repertoire of cryptic secondary metabolite biosynthetic gene clusters (BGCs), is extensively exploited as a microbial chassis for heterologous biosynthesis. Mining of its secondary metabolites facilitates the discovery of novel bioactive compounds and the development and application of chassis cells. In the course of heterologous expression of exogenous genes in *A. nidulans*, we unexpectedly observed the activation of cryptic host BGCs, which resulted in substantial alterations to its secondary metabolic profile. Four previously undescribed compounds (**1**–**4**), together with six known analogs (**5**–**10**), were isolated from three recombinant *A. nidulans* strains. Notably, compounds **1**–**3** are the first naturally occurring examples of diketopiperazine–isoindolinone hybrid alkaloids, while compound **4** is a previously unreported benzofuran carboxylic acid derivative. Their structures and absolute configurations were assigned by interpretation of a combination of spectroscopic data and electronic circular dichroism calculations. Compounds **4** and **5** exhibited potent DPPH radical scavenging activity (IC_50_, 6.01 and 7.00 μg·mL^−1^, respectively). This study uncovers a “metabolic perturbation” effect on the host metabolic network during heterologous expression and offers a new strategy for activating silent gene clusters and discovering novel natural products through genetic manipulation.

## 1. Introduction

*Aspergillus nidulans* is a classic type strain of filamentous fungi and holds an irreplaceable position in the advancement of modern synthetic biology and microbial natural product discovery [1]. Its superiority is attributed to several core characteristics: first, it harbors a highly well-characterized and sophisticated genetic manipulation system, encompassing high-efficiency transformation protocols, a diverse array of selectable markers, and stringently controllable promoters, which enable precise and efficient gene knockout, overexpression, and heterologous integration. Second, *A. nidulans* itself has a strong and comprehensive biosynthetic capacity for secondary metabolites; its genome contains dozens of gene clusters encoding polyketide synthases (PKSs), non-ribosomal peptide synthetases (NRPSs), and hybrid enzymes, representing a vast reservoir of natural products. More importantly, as a eukaryotic expression system, *A. nidulans* possesses post-translational modification, folding, and secretion pathways similar to those of higher organisms, enabling correct expression and assembly of multienzyme complexes with complex structures, making it outperform prokaryotic systems [2]. Therefore, *A. nidulans* has been widely engineered as an efficient “cell factory” for heterologous production of pharmaceutical precursors (e.g., penicillin [3]), industrial enzymes (e.g., cellulase [4]), and high-value natural products. Systematic exploration of the secondary metabolic profile of *A. nidulans* not only deepens the research dimension of this type strain to promote its development and application as a cell factory but also is a key approach to discovering structurally novel and biologically active natural products [5].

**Figure 1:**
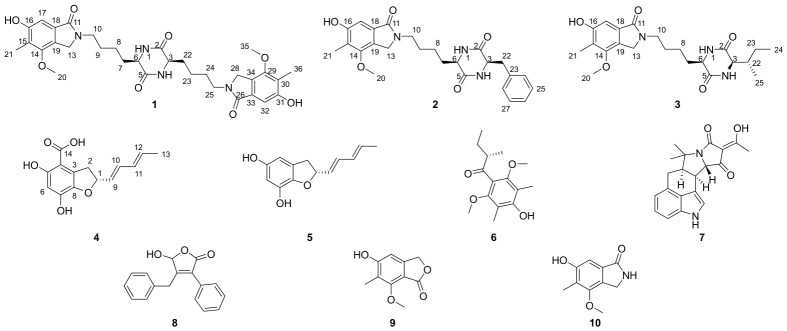
Chemical structures of compounds **1**–**10**.

Heterologous expression of silent biosynthetic gene clusters (BGCs) represents a key strategy for the discovery of novel bioactive natural products. In this study, we observed the unexpected activation of host silent BGCs in *A. nidulans* during heterologous expression [6,7]. When introducing two exogenous BGCs via an auxotrophic plasmid into *A. nidulans*, the secondary metabolic profiles of the corresponding recombinant strains were significantly altered. Then, we targeted the isolation of the newly generated metabolites from liquid cultures of the recombinant strains. From the ethyl acetate extract of the fermentation broth, we successfully isolated and identified ten compounds (Figure 1), including four new compounds (**1**–**4**) and six known compounds (**5**–**10**). Compounds **1**–**3** are the first naturally occurring examples of diketopiperazine–isoindolinone hybrid alkaloids. Compound **4** is a previously unreported benzofuran carboxylic acid derivative. And compounds **5**–**7** were isolated from *A. nidulans* for the first time. Analysis of the structures and biogeneses of the isolated compounds revealed that the biosynthesis of these compounds was not associated with exogenous genes but attributed to the activation of silent gene clusters in the host. In addition, the in vitro antioxidant activities of all compounds were preliminarily evaluated, and compounds **4** and **5** showed significant DPPH radical scavenging activities.

## 2. Materials and Methods

### 2.1. General Experimental Procedures

Optical rotations were measured on an AUTOPOL IV polarimeter (Rudolph Research Analytical, Hackettstown, NJ, USA). CD spectra were recorded on a JASCO J-815 circular dichroism spectrometer (Jasco, Hachioji, Japan). ^1^H, ^13^C NMR, and 2D NMR spectra were recorded on Bruker AV-III-500 and Bruker AV III-700 HD NMR spectrometers (Bruker, Rheinstetten, Germany) using tetramethylsilane (TMS) as a reference. ESI-MS and analytical HPLC were taken on a Waters ACQUITY H-Class with QDA mass detector (Kinetex^®^ C18 column, 1.7 μm, 100 mm × 2.1 mm, Phenomenex, Torrance, CA, USA). HR-ESI-MS data were performed on an Agilent 6520 Accurate-Mass Q-TOFLC/MS spectrometer (Agilent Technologies, Ltd., Santa Clara, CA, USA). Reversed-phase medium-pressure liquid chromatography (RP-MPLC) was carried out using the BUCHI Reveleris^®^ Prep medium-pressure liquid chromatography system (Büchi, Flawil, Switzerland); semi-preparative reversed-phase HPLC was performed on an SSI semi-preparative HPLC system with a DAD detector (Scientific Systems, State College, PA, USA) using a Silgreen C18 column (5 μm, 250 mm × 20 mm, Beijing Green Herbs Science and Technology, Beijing, China).

### 2.2. Materials and Culture Conditions

The strains used in this study are listed in Table S1. *Penicillium dangeardii* and *Calcarisporium arbuscula*, the source fungi of the exogenous gene clusters, were cultured on potato dextrose agar (PDA, BD) at 28 °C for 5–7 days. Mycelia were then transferred into potato dextrose broth (PDB, BD) for genomic DNA extraction. *Escherichia coli* XL1-Blue was used for DNA manipulation. The recombinant *A. nidulans* strains used in this study were constructed by transforming the auxotrophic host strain A8030 [8] with expression plasmids carrying the target biosynthetic genes, following the general methods described by Wang et al. [9]. The expression cassettes utilized four different promoters to drive gene expression: the constitutive *gpdA* promoter, the inducible *AmyB* promoter, the *glaA* promoter and the *Ptub* promoter. Selection markers complementing uracil (*pyrG*), pyridoxine (*pyroA*), and riboflavin (*riboB*) auxotrophies were employed to isolate transformants. *Saccharomyces cerevisiae* BJ5464-NpgA was used for plasmid construction.

### 2.3. Bioinformatic Analysis

The whole-genome genomes of *P. dangeardii* [10,11] and *C. arbuscula* [12,13] had been previously sequenced. The gene clusters were analyzed by antiSMASH fungal version 7.0 “https://fungismash.secondarymetabolites.org” (accessed on 21 March 2025) and 2ndFind “https://biosyn.nih.go.jp/2ndfind” (accessed on 28 March 2025), manually checked by NCBI blast “https://www.ncbi.nlm.nih.gov/” (accessed on 16 April 2025).

### 2.4. Gene Cloning, Plasmid Construction, and Genetic Manipulation

The plasmids and primers used in this work are listed in Tables S2 and S3, respectively. After 4 days of culturing in PDB, the genomic DNA of *P. dangeardii* and *C. arbuscula* was extracted using a genomic DNA extraction kit (Tiangen, Beijing, China). All *nrp* and *tca* genes and their native terminators (500 bp downstream from the stop codon) were amplified by PCR using genomic DNA as the template. Polymerase chain reactions for cloning were performed using KOD-plus-neo or KOD one^TM^ PCR Master Mix (Toyobo Life Science, Osaka, Japan) according to the manufacturer’s instructions. DNA restriction enzymes were used as recommended by the manufacturer (NEB, Ipswich, MA, USA). The plasmids for *A. nidulans* A8030 expression were constructed by the yeast homologous recombination method. Briefly, these pCR fragments and linearized pANP/pANR/pANU were co-transformed to *S. cerevisiae* using the PEG/LiAc-mediated method and spread on histidine–uracil dropout semisynthetic medium. The plasmids of single clones were rescued using the Zymoprep Yeast Miniprep Kit (Zymo Research, Irvine, CA, USA) and transformed to *E. coli* XL1-Blue for sequencing. The same procedures were used for the construction of all plasmids. Constructs verified by restriction enzyme digestion and Sanger sequencing were used for *A. nidulans* transformation.

### 2.5. Transformation of A. nidulans

Protoplasts were prepared via enzymatic digestion of germinated conidia using a combination of lysing enzyme (Sigma-Aldrich, St. Louis, MO, USA) and Yatalase (Takara, Shiga, Japan) in Osmetic Buffer. Purified plasmid DNA (5–10 μg) was added to the protoplast suspension in STC Buffer, and the mixture was incubated on ice for 50 min. PEG-mediated transformation was performed by the addition of PEG4000 solution, after which the transformation mixture was plated onto selective CD-sorbitol medium. Transformants were purified through single-colony isolation and subsequently verified by diagnostic PCR. For multi-gene expression, either individual plasmids or co-transformation of multiple expression cassettes was carried out. Correct integration of the expression constructs was confirmed by PCR amplification using primers specific to the flanking genomic regions [14]. At last, three recombinant strains, AN-*nrpACDEFGHI*, AN-*nrpEFGHI* and AN-*tcaABCDEFGHI,* were constructed. Transformants were verified by PCR, transferred to fresh plates and cultured for 2–3 days; then spores were harvested and stored at −80 °C. To verify whether the exogenous genes were transcribed, reverse transcription-PCR (RT-PCR) assays of six representative genes (*tcaA*, *tcaB*, *tcaF*–*tcaI*) from the recombinant strain AN-*tcaABCDEFGHI* were performed. The RT-PCR method and results were provided in the Supporting Information (Figure S23). The results demonstrated that the tested heterologous genes were actively transcribed in the host strain.

### 2.6. Fungal Fermentation

Spores of the three *A. nidulans* recombinant strains stored at −80 °C, were evenly plated onto CD sporulation medium and activated via incubation at 37 °C for 48 h. The activated spores were subsequently inoculated into 1 L of CD-ST liquid fermentation medium in Erlenmeyer flasks, with a total fermentation volume of 6 L for each of the three *A. nidulans* recombinant strains, followed by incubation at 25 °C with orbital shaking at 220 rpm for 7 days.

### 2.7. Extraction and Isolation

Following completion of the fermentation, the culture broth was mixed with an equal volume of ethyl acetate and subjected to thorough liquid–liquid extraction via vigorous shaking; this extraction procedure was repeated three times. The combined ethyl acetate fractions were concentrated under reduced pressure at 40 °C, yielding dark brown crude extract 1 (1.2 g, from AN-*nrpACDEFGHI*), dark brown crude extract 2 (1.3 g, from AN-*tcaABCDEFGHI*), and dark brown crude extract 3 (1.5 g, from AN-*nrpEFGHI*), respectively.

Extract 1 was mixed with 2.5 g of diatomaceous earth and subjected to ODS medium-pressure liquid chromatography with a gradient of methanol–water (30–99%, *v*/*v*) to obtain fractions 1–6 (R1–R6). Fractions containing target compounds were R5 (32.90 mg). R5 was separated by semi-preparative HPLC (methanol–water, 28:72, *v*/*v*, 0.1% formic acid, Silgreen column, 250 mm × 20 mm) at 3.0 mL/min to yield compound **1** (3.50 mg, *t*_R_ = 18.7 min), compound **2** (3.00 mg, *t*_R_ = 16.1 min), and compound **3** (2.50 mg, *t*_R_ = 13.5 min).

Extract 2 was mixed with 3 g of diatomaceous earth and subjected to ODS medium-pressure liquid chromatography with a gradient of acetonitrile–water (40–99%, *v*/*v*) to obtain fractions 1–9 (C1–C9). Fractions containing target compounds were C3 (20.10 mg), C4 (8.50 mg) and C7 (116.30 mg). C3 and C4 were analyzed by LC-MS (acetonitrile–water, 5–99%, *v*/*v*) and found to be pure (>95%); thus, they were not further purified and were identified as compound **4** (8.50 mg, *t*_R_ = 5.10 min) and compound **5** (20.10 mg, *t*_R_ = 4.65 min), respectively. C7 was separated by semi-preparative HPLC (acetonitrile–water, 50:50, *v*/*v*, 0.1% formic acid, Silgreen column, 250 mm × 20 mm) at 3.0 mL/min to yield compound **6** (2.64 mg, *t*_R_ = 28.5 min), compound **7** (13.14 mg, *t*_R_ = 31.6 min), and compound **8** (4.43 mg, *t*_R_ = 35.9 min).

Extract 3 was mixed with 4 g of diatomaceous earth and subjected to ODS medium-pressure liquid chromatography with a gradient of methanol–water (30–99%, *v*/*v*) to obtain fractions 1–6 (U1–U6). Fractions containing target compounds were U2 (58.00 mg). U2 was separated by semi-preparative HPLC (methanol–water, 28:72, *v*/*v*, 0.1% formic acid, Silgreen column, 250 mm × 20 mm) at 3.0 mL/min to yield compound **9** (1.00 mg, *t*_R_ = 12.5 min) and compound **10** (1.50 mg, *t*_R_ = 14.5 min).

Cicpiperazinone A (**1**): pale yellow powder; sparingly soluble in methanol; ${\left[\alpha \right]}_{D}^{20}$ -35.7 (*c* 0.042, MeOH); ^1^H and ^13^C NMR data, see Table 1 and Figures S1–S5; HR-ESI-MS showed a quasi-molecular ion peak at *m*/*z* 609.2929 [M+H]^+^ (calcd for C_32_H_41_N_4_O_8_, 609.2919), indicating a molecular formula of C_32_H_40_N_4_O_8_ with 15 degrees of unsaturation.

Cicpiperazinone B (**2**): pale yellow powder; slightly soluble in methanol; ${\left[\alpha \right]}_{D}^{20}$ -26.1 (*c* 0.092, MeOH); ^1^H and ^13^C NMR data, see Table 1 and Figures S6–S10; HR-ESI-MS showed a quasi-molecular ion peak at *m*/*z* 452.2187 [M+H]^+^ (calcd for C_25_H_30_N_3_O_5_, 452.2180), indicating a molecular formula of C_25_H_29_N_3_O_5_ with 13 degrees of unsaturation.

Cicpiperazinone C (**3**): pale yellow powder; slightly soluble in methanol; ${\left[\alpha \right]}_{D}^{20}$ -31.5 (*c* 0.108, MeOH); ^1^H and ^13^C NMR data, see Table 1 and Figures S11–S15; HR-ESI-MS showed a quasi-molecular ion peak at *m*/*z* 418.2340 [M+H]^+^ (calcd for C_22_H_32_N_3_O_5_, 418.2336), indicating a molecular formula of C_22_H_31_N_3_O_5_ with 9 degrees of unsaturation.

Asperfuranic acid (**4**): brown-red amorphous powder; readily soluble in methanol;${\left[\alpha \right]}_{D}^{20}$ -12.62 (*c* 0.103, MeOH); ^1^H and ^13^C NMR data, see Table 2 and Figures S16–S21; HR-ESI-MS showed a quasi-molecular ion peak at *m*/*z* 263.0923 [M+H]^+^ (calcd for C_14_H_15_O_5_, 263.0914), indicating a molecular formula of C_14_H_14_O_5_ with 8 degrees of unsaturation.

### 2.8. ECD Calculations of **1** and **2**

Conformational analyses of compounds **1** and **2** were performed in the Yinfo Cloud Platform (https://cloud.yinfotek.com) using Confab [15] with the stochastic algorithm at an MMFF94 force field with an RMSD threshold of 0.5 Å and an energy window of 7 kcal·mol^−1^. All theoretical calculations were carried out using Gaussian 09 (Revision D.01). Conformers were consecutively optimized at the PM6 semi-empirical level and HF/6-31G(d) theoretical level, and room-temperature equilibrium populations were calculated based on the Boltzmann distribution law, with dominant conformers showing populations greater than 1% retained for further optimization. The selected conformers were finally fully optimized at the B3LYP/6-31G(d) level in the gas phase, and vibrational frequency analysis was performed to confirm no imaginary frequencies for all stable structures. ECD calculations were conducted in methanol with the IEFPCM solvation model via time-dependent density functional theory (TD-DFT) at the B3LYP/6-311G(d,p) level, with rotatory strengths for 30 excited states calculated for each conformer. The final ECD spectra were simulated by overlapping Gaussian functions for each transition using the ECD/UV analysis tool in the Yinfo Cloud Platform and were compared with the experimental CD spectra.

### 2.9. Bioactivity Assay

The in vitro antioxidant activities of compounds **1**–**10** were evaluated using the 1,1-diphenyl-2-picrylhydrazyl (DPPH) radical scavenging method [16], with the half-maximal inhibitory concentration (IC_50_) as the evaluation index and ascorbic acid (Vc) as the positive control. Experimental procedure: Compounds **1**–**10** and Vc were dissolved in anhydrous methanol to prepare 10.0 mM stock solutions, which were serially diluted to 250, 125, 62.5, 31.25, and 15.63 μM test solutions. DPPH was dissolved in anhydrous ethanol to prepare a working solution at 200 μM. In 1.5 mL Eppendorf tubes, experimental groups (A_i_: 150 μL sample solution + 150 μL DPPH working solution), control groups (A_c_: 150 μL sample solution + 150 μL anhydrous methanol), and blank groups (Aⱼ: 150 μL anhydrous methanol + 150 μL DPPH working solution) were set up, each concentration in triplicate. After mixing, the tubes were incubated at room temperature in the dark for 30 min. After the reaction, 200 μL of each reaction mixture was transferred to a 96-well plate, and the absorbance was measured at 517 nm using a microplate reader. The DPPH radical scavenging rate, K (%), was calculated as K (%) = [1 − (A_i_ − A_c_)/A_j_] × 100%, where A_i_, A_c_, and A_j_ are the absorbance values of the corresponding groups. Data were analyzed using Microsoft Excel; the half-logarithmic curve of scavenging rate versus concentration was plotted, and the IC_50_ was calculated by a cubic regression equation to compare and evaluate the antioxidant activities of the compounds.

## 3. Results

### 3.1. Heterologous Expression Products Analysis

**Figure 2:**
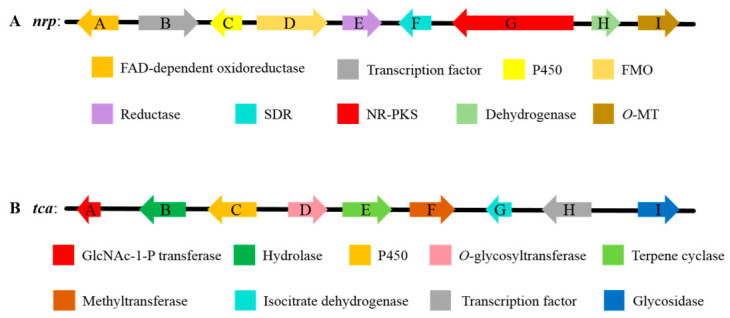
(**A**) The NR-PKS BGC *nrp* from *P. dangeardii*. The core gene of this gene cluster is NR-PKS *nrpG*. Its domain analysis revealed a modular architecture consisting of starter unit acyltransferase (SAT), acyltransferase (AT), ketosynthase (KS), product template (PT), acyl carrier protein (ACP), and thioesterase (TE) domains. Flanking *nrpG* are genes encoding auxiliary enzymes and regulatory elements, including a FAD-dependent oxidoreductase (NrpA), a transcription factor (NrpB), a cytochrome P450 enzyme (NrpC), a flavin-containing monooxygenase (NrpD), a reductase (NrpE), a short-chain dehydrogenase (NrpF), a dehydrogenase (NrpH), and an O-methyltransferase (NrpI). (**B**) The sesquiterpene BGC *tca* from *C. arbuscula*. The core gene of this gene cluster is the terpene cyclase *tcaE*. Other enzymes include a GlcNAc-1-P transferase (TcaA), a hydrolase (TcaB), a cytochrome P450 enzyme (TcaC), an O-glycosyltransferase (TcaD), a methyltransferase (TcaF), an isocitrate dehydrogenase (TcaG), a transcription factor (TcaH), and a glycosidase (TcaI).

**Figure 3:**
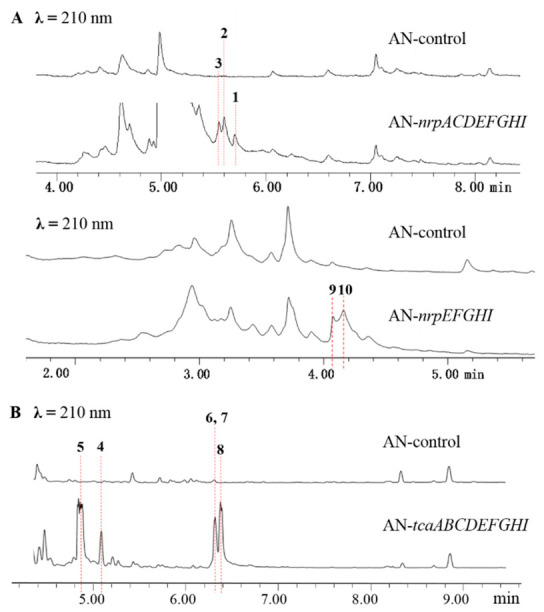
HPLC analyses of the crude extracts from the recombinant strains of (**A**) the NR-PKS BGC *nrp* and (**B**) the sesquiterpene BGC *tca*.

To discover structurally novel natural products, we mined two putative BGCs (Figure 2), including a non-reducing polyketide synthase (NR-PKS) BGC (*nrp*) and a sesquiterpene BGC (*tca*) from two previously analyzed endophytic fungi, *P. dangeardii* [10,11] and *C. arbuscula* [12,13], respectively, for heterologous expression. We used *A. nidulans* A8030 as the heterologous host and created three recombinant strains: AN-*nrpACDEFGHI,* AN-*nrpEFGHI* and AN-*tcaABCDEFGHI*. Comparative chromatographic analysis of the crude extracts from the recombinant strains and the control strain (transformed with the corresponding empty expression vectors containing the selection markers and promoters without any exogenous genes) showed that their secondary metabolic profiles displayed significant differences (Figure 3). All three recombinant strains generated a series of new product peaks that were not observed in the control strain, suggesting that cryptic BGCs in the three recombinant strains had been activated. To characterize the newly produced metabolites in the recombinant strains, we conducted large-scale fermentation and performed metabolite isolation from the fermentation extract of the recombinant strains. As a result, three novel diketopiperazine–isoindolinone hybrid alkaloids, cicpiperazinones A–C (**1**–**3**), two known polyketides, nidulol (**9**) and cichorine (**10**), were isolated from the recombinant strains AN-*nrpACDEFGHI* and AN-*nrpEFGHI* of an NR-PKS BGC *nrp*, respectively. Four polyketides, including a new asperfuranic acid (**4**), asperfuran (**5**), dichotomone (**6**), and microperfuranone (**8**), as well as one PKS-NRPS hybrid *α*-cyclopiazonic acid (**7**), were isolated from the recombinant strain AN-*tcaABCDEFGHI* of a sesquiterpene BGC *tca*.

Further analyzing the correlation between the newly produced compounds and the exogenous gene clusters, we obtained unexpected results. The BGC *cic* of compounds **9**–**10**, differing from the exogenous BGC *nrp*, had been previously found in *A. nidulans* [17]. Compounds **1**–**3** are diketopiperazine derivatives incorporating a fragment of compound **10**. Thus, the biosynthesis of these compounds does not originate from the exogenous NR-PKS BGC *nrp* but is more possibly related to the activation of silent BGCs in host *A. nidulans*. Meanwhile, compounds **4**–**8** belong to polyketides or PKS-NRPS hybrids, obviously indicating that the biosynthesis of them is not related to the sesquiterpene BGC *tca*. Collectively, structure and biosynthesis correlation analysis suggested that the biosynthesis of **1**–**10** was not derived from the heterologously expressed BGCs. Instead, all isolated compounds were related to the activation of cryptic BGCs endogenous to the host *A. nidulans*.

### 3.2. Structure Elucidation

Compound **1**: pale yellow powder, sparingly soluble in methanol, ${\left[\alpha \right]}_{D}^{20}$ -35.7 (*c* 0.042, MeOH). HR-ESI-MS showed a quasi-molecular ion peak at *m*/*z* 609.2929 [M+H]^+^ (calcd for C_32_H_41_N_4_O_8_, 609.2919), indicating a molecular formula of C_32_H_40_N_4_O_8_ with 15 degrees of unsaturation. The ^1^H NMR (700 MHz, DMSO-*d_6_*) and ^13^C NMR (175 MHz, DMSO-*d_6_*) spectra (Table 1), in conjunction with the molecular formula, suggested that **1** is a symmetric dimer, with half of the molecule accounting for 16 carbons. The ^13^C NMR spectrum displayed signals for 8 sp^2^ carbons (including two carbonyls) and 8 sp^3^ carbons per half. Detailed analysis of the 1D NMR data with the aid of an HSQC spectrum revealed the presence of two exchangeable protons [*δ*_H_ 8.12 (s, 1-NH) and 9.77 (s, 16-OH)], two methines [*δ*_C_ 103.0 (C-17), *δ*_C_ 53.9 (C-6)], five methylenes [*δ*_C_ 47.9 (C-13), 41.5 (C-10), 32.8 (C-7), 27.6 (C-9), 21.8 (C-8)], and two methyl groups [*δ*_C_ 58.8 (C-20), 9.4 (C-21)].

**Figure 4:**
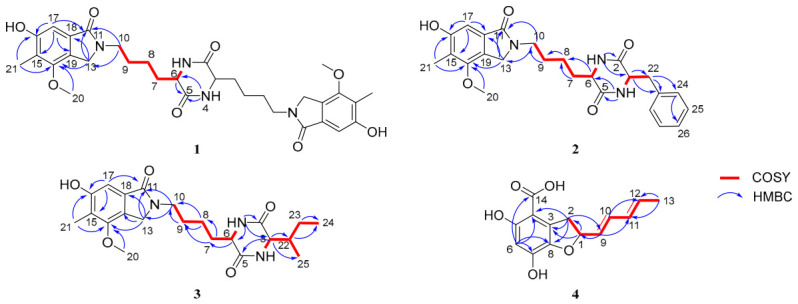
^1^H-^1^H COSY and key HMBC correlations of compounds **1**–**4**.

The ^1^H-^1^H COSY spectrum (Figure 4) revealed a continuous aliphatic chain along with an N-atom spin system for 1-NH/H-6/H_2_-7/H_2_-8/H_2_-9/H_2_-10, establishing the N1-C10 chain. HMBC correlations (Figure 4) were pivotal in assembling the structure. Correlations from H-6 to C-5/C-1 (*δ*_C_ 167.9), C-7, and C-8, and from 4-NH to C-5 and C-6, established the presence of a 2,5-diketopiperazine (DKP) ring. The remaining fragment was identified as a highly substituted isoindolinone moiety. HMBC correlations from H-10 to C-8, C-9, C-11 (*δ*_C_ 167.1), and C-13 (*δ*_C_ 47.9); from H-13 to C-11, C-14 (*δ*_C_ 153.3), C-18 (*δ*_C_ 120.7), and C-19 (*δ*_C_ 131.9); and from H-17 (*δ*_H_ 6.82) to C-11, C-15 (*δ*_C_ 118.9), C-16 (*δ*_C_ 156.5), and C-19, combined with the chemical shifts in C-10 and C-13, confirmed a 2,3-dihydro-isoindolin-1-one core with the N-atom connected to C-10. The placements of the methoxy, methyl, and hydroxyl groups on this core were deduced from HMBC correlations: H_3_-20 (*δ*_H_ 3.87) with C-14, H_3_-21 (*δ*_H_ 2.05) with C-14, C-15, and C-16, and the exchangeable proton 16-OH with C-15. Thus, the planar structure of **1** was elucidated as a symmetric diketopiperazine dimer consisting of a DKP core with two identical 4-(isoindolinone)butyl side chains.

**Figure 5:**
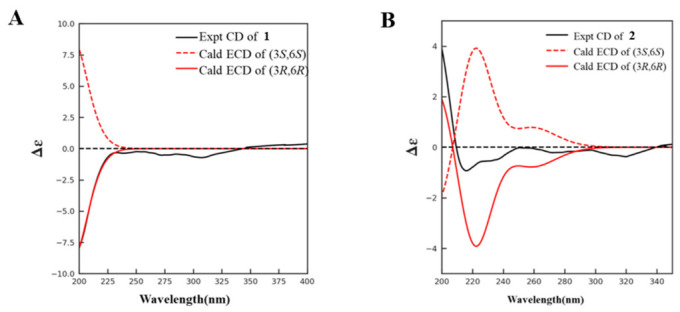
Experimental and calculated ECD spectra of compounds (**A**) **1** and (**B**) **2**.

Compound **1** possesses two chiral centers. Its optical rotation is ${\left[\alpha \right]}_{D}^{20}$ -35.7 (*c* 0.042, MeOH). If the relative configuration was (R*, S*), the compound would be a mesomer and optically inactive. Therefore, the absolute configuration of **1** must be either (3*S*, 6*S*) or (3*R*, 6*R*). Comparison of the experimental CD spectrum of compound **1** in comparison with calculated ECD spectra (Figure 5A) showed that the experimental CD spectrum of **1** was in good agreement with that of the (3*R*, 6*R*) enantiomer. Literature reports [18] indicated that diketopiperazines with the (3*S*, 6*S*) configuration exhibit positive optical rotation, opposite to that of compound **1**, further supporting the (3*R*, 6*R*) assignment. Consequently, the absolute configuration of **1** was assigned as (3*R*, 6*R*), and it was named cicpiperazinone A.

Compound **2**: pale yellow powder, slightly soluble in methanol, ${\left[\alpha \right]}_{D}^{20}$ -26.1 (*c* 0.092, MeOH). HR-ESI-MS showed a quasi-molecular ion peak at *m*/*z* 452.2187 [M+H]^+^ (calcd for C_25_H_30_N_3_O_5_, 452.2180), indicating a molecular formula of C_25_H_29_N_3_O_5_ with 13 degrees of unsaturation. Comparison of the NMR data of **2** (Table 1) with those of **1** revealed a high degree of similarity for signals corresponding to the isoindolinone moiety (C-11 to C-21) and part of the aliphatic chain (C-6 to C-10), suggesting that **2** shares the same 4-(isoindolinone)butyl side chain as **1**. However, significant differences were observed in the DKP ring region. Instead of the symmetric DKP signals, the NMR spectra of **2** displayed resonances for an extra monosubstituted benzene ring [*δ*_C_ 136.2 (C-23), 130.4 (C-24), 128.0 (C-25), 126.7 (C-26); *δ*_H_ 7.14–7.22] and an additional methylene (*δ*_C_ 38.2, C-22), along with the asymmetric DKP ring [*δ*_C_ 55.3 (C-3), 53.7 (C-6), 167.0 (C-2), 166.2 (C-5); *δ*_H_ 8.05 (s, 1-NH), 8.12 (s, 4-NH)]. This indicated the presence of a phenylalanine, which was confirmed by HMBC (Figure 4) correlations from H-3 to C-22 and C-23, and from H_2_-22 to C-2, C-3, and the aromatic carbons C-23 and C-24. Therefore, the planar structure of **2** was established as a DKP with a benzyl substituent at C-3 and the same 4-(isoindolinone)butyl side chain at C-6 as in **1**.

**Figure 6:**
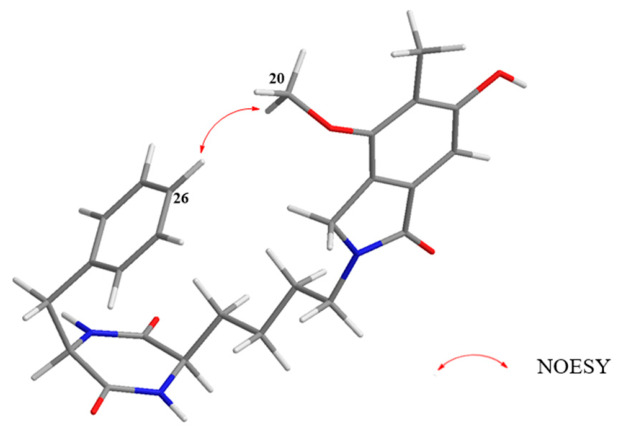
Key NOESY correlation of compound **2**.

The relative configuration of compound **2** was established by analysis of the NOESY spectrum. The key NOE correlation (Figure 6) between H_3_-20 and H-26 indicated that the benzyl and the 4-(isoindolinone)butyl side chain are situated on the same face of the DKP ring. Consequently, the absolute configuration of compound **2** is restricted to either (3*R*, 6*R*) or (3*S*, 6*S*). The experimental CD spectrum of **2** matched well with the calculated ECD spectrum for the (3*R*, 6*R*) enantiomer (Figure 5B), which was consistent with the negative optical rotation (${\left[\alpha \right]}_{D}^{20}$ -26.1) and the biosynthetic relationship with **1**. Thus, the absolute configuration of **2** was also assigned as (3*R*, 6*R*), and compound **2** was named cicpiperazinone B.

Compound **3**: pale yellow powder, slightly soluble in methanol, ${\left[\alpha \right]}_{D}^{20}$ -31.5 (*c* 0.108, MeOH). HR-ESI-MS showed a quasi-molecular ion peak at *m*/*z* 418.2340 [M+H]^+^ (calcd for C_22_H_32_N_3_O_5_, 418.2336), indicating a molecular formula of C_22_H_31_N_3_O_5_ with 9 degrees of unsaturation. The NMR data of **3** (Table 1) were very similar to those of **2**, particularly for the signals corresponding to the isoindolinone moiety (C-11 to C-21) and the aliphatic chain (C-6 to C-10). The key difference was the replacement of the benzyl group signals in **2** with signals for an isobutyl group [*δ*_C_ 38.0 (C-22), 24.3 (C-23), 15.1 (C-24), 11.9 (C-25); *δ*_H_ 1.83 (H-22), 1.39 (H_2_-23), 0.89 (H_3_-24), 0.83 (H_3_-25)].

The ^1^H-^1^H COSY spectrum showed correlations for H_3_-25/H-22/H_2_-23/H_3_-24, confirming the presence of the isobutyl fragment. HMBC correlations from H-3 to C-2, C-22, and C-25, and from H-22 to C-2, C-24, and C-25 positioned the isobutyl group at C-3 of the DKP ring (Figure 4). Thus, the planar structure of **3** was established as a DKP with an isobutyl substituent at C-3 and the same 4-(isoindolinone)butyl side chain at C-6.

Based on the same biogenetic considerations as **1** and **2** and the negative optical rotation value (${\left[\alpha \right]}_{D}^{20}$ -31.5), which was consistent with **1** and **2**, the absolute configuration at C-3 and C-6 was proposed to be 3*R*,6*R*. The configuration at C-22 was assigned as *S*, in accordance with that of *L-*isoleucine, a biosynthetic precursor of **3**. Thus, the absolute configuration of **3** was putatively assigned as 3*R*,6*R*,22*S*, and compound **3** was named cicpiperazinone C.

Compound **4**: brown-red amorphous powder, readily soluble in methanol, ${\left[\alpha \right]}_{D}^{20}$ -12.6 (*c* 0.103, MeOH). HR-ESI-MS showed a quasi-molecular ion peak at *m*/*z* 263.0923 [M+H]^+^ (calcd for C_14_H_15_O_5_, 263.0914), indicating a molecular formula of C_14_H_14_O_5_ with 8 degrees of unsaturation. ^1^H NMR (500 MHz, CD_3_OD) and ^13^C NMR (125 MHz, CD_3_OD) data are shown in Table 2. The ^13^C NMR spectrum displayed 11 sp^2^ signals (including one carbonyl) and three sp^3^ signals. Analysis of the HSQC spectrum identified one methyl (*δ*_C_ 18.3, C-13), one methylene (*δ*_C_ 28.5, C-2), six methines (five olefinic and one oxygenated), and six quaternary carbons (five aromatic and one carboxylic *δ*_C_ 171.8, C-14).

The ^1^H-^1^H COSY spectrum (Figure 4) revealed a heptadiene spin system (H_2_-2/H-1/H-9/H-10/H-11/H-12/H_3_-13). The large coupling constants (*J*_9,10_ = 15.3 Hz and *J*_11,12_ = 14.5 Hz) established the trans geometry for both the Δ^9^ and Δ^11^ double bonds. HMBC correlations (Figure 4) from H-6 to C-8 (*δ*_C_ 135.9), C-7 (*δ*_C_ 155.7) and C-4 (*δ*_C_ 100.2), from H_2_-2 to C-3 (*δ*_C_ 125.6), C-4, and C-8, and from H-1 to C-2, and C-3, combined the downfield chemical shifts in C-1 (*δ*_C_ 80.65) and C-8, established the 2,3-dihydrobenzofuran ring connected with the conjugated diene side chain at C-1. The highly downfield-shifted quaternary carbons C-5 (*δ*_C_ 159.1) and C-7 (*δ*_C_ 155.7) indicated they were oxygenated, and the chemical shift in C-6 (*δ*_C_ 101.9) was characteristic of a carbon in benzene ring flanked by two oxygenated substituents, confirming the presence of two hydroxyl groups at C-5 and C-7. The carboxylic acid group C-14 (*δ*_C_ 171.8) was placed at C-4 based on HMBC correlation from H-6 to C-4 and C-14. Furthermore, the Infrared (IR) spectrum of compound **4** displayed characteristic absorptions of a carboxylic acid functionality, including a broad O-H stretching band (3300–2500 cm^−1^) and a carbonyl absorption (1673 cm^−1^) of a conjugated carboxyl group, thus corroborating the presence of a free carboxyl moiety (Figure S22).

Since the planar structure of compound **4** is highly similar to the known compound asperfuran, just possessing an additional carboxyl group at C-4, and the optical rotation of compound **4** (${\left[\alpha \right]}_{D}^{20}$ -12.6) has the same sign as that of (*R*)-asperfuran [19] (${\left[\alpha \right]}_{D}^{20}$ -20.9), the absolute configuration of C-1 in **4** was assigned as *R*. Then, compound **4** was named asperfuranic acid.

In addition to the above new compounds (**1**–**4**), six known compounds were identified as asperfuran (**5**) [19], dichotomone (**6**) [20,21], *α*-cyclopiazonic acid (**7**) [22,23], microperfuranone (**8**) [24,25], nidulol (**9**) [17], cichorine (**10**) [17,26], respectively, by comparing spectroscopic and specific rotation data with those reported in the literature. This represents the first report of compounds **5**–**7** from *A. nidulans*.

### 3.3. Bioactivity Assay

The in vitro antioxidant activities of compounds **1**–**10** were preliminarily evaluated using the DPPH radical scavenging method. The results demonstrated that compounds **4** and **5** exerted potent free radical scavenging activity, whereas compounds **6**–**8** displayed moderate antioxidant efficacy (Table 3).

## 4. Discussion

In this study, during heterologous expression in *A. nidulans*, we unexpectedly observed the activation of silent secondary metabolite gene clusters from the host strain, leading to the isolation of ten newly produced metabolites, including three novel diketopiperazine–isoindolinone hybrids (**1**–**3**) and one new benzofuran carboxylic acid derivative (**4**). This phenomenon suggests that introduction of exogenous genes is not a simple “addition” process but may trigger extensive remodeling of metabolic and regulatory networks within the host cell. From a mechanistic perspective, heterologous expression might perturb the host and activate silent gene clusters through the following pathways:

(i) Perturbation of intracellular metabolite homeostasis [27]: The expression of heterologous enzymes may lead to the consumption of specific biosynthetic precursors (e.g., acetyl-CoA, malonyl-CoA, or farnesyl pyrophosphate). This imbalance would alter metabolic flux, and the host might respond by activating cryptic BGCs to compensate or rebalance precursor pools. A metabolomics approach, tracking global changes in primary and secondary metabolites, would be the most direct way to test this hypothesis.

(ii) Competition for regulatory proteins [6]: The heterologous expression vectors may occupy transcriptional or translational resources, indirectly affecting the expression and function of endogenous regulators, leading to activation of some silent gene clusters. The strong constitutive or inducible promoters used in our constructs could competitively acquire essential transcription factors or RNA polymerases. This resource competition might disrupt the delicate balance of repressors and activators that normally maintain chromatin in a silent state. We speculate that this could particularly affect clusters under the control of global regulators like *LaeA* [28]. This possible mechanism can be tested via RNA-Seq to compare the global transcriptomic landscape of the recombinant strains versus the control strain, specifically seeking signatures of transcriptional machinery overload and the coordinated upregulation of whole BGCs, which is also helpful for the discovery of cryptic BGCs.

(iii) Stress response activation [29]: Introduction of foreign DNA or expression of heterologous proteins may trigger cellular stress responses in fungi, activating various defense mechanisms including secondary metabolism. Fungi possess conserved stress-sensing pathways (e.g., cell wall integrity pathway, HOG-MAPK pathway). The act of plasmid integration via protoplast transformation, followed by the production of exogenous proteins, could activate these pathways, which in turn are known to cross-talk with secondary metabolism. For instance, the upregulation of antioxidant compounds like **4** and **5** might represent a direct response to heterologous protein-induced oxidative stress. A comparative metabolome and transcriptome analysis would help distinguish between a specific pathway activation and a general stress response.

(iv) Epigenetic regulation perturbation [30]: Studies have shown that expression of secondary metabolite gene clusters in fungi is often regulated by epigenetic mechanisms such as histone modifications. Heterologous expression may interfere with the function or expression balance of related epigenetic modifying enzymes. The import of a large expression construct into *A. nidulans* could alter local chromatin architecture or interfere with the function of histone-modifying enzymes. This might inadvertently open up condensed heterochromatic regions where many silent BGCs reside, making them accessible to the transcriptional machinery. While we did not perform such experiments here, future studies employing ATAC-seq (to assess chromatin accessibility) or ChIP-seq [31] using antibodies against modified histones (e.g., H3K9me3, H3K14ac) would be the definitive approach to test the role of epigenetic regulation in this perturbation effect.

In addition, compounds **1**–**3** possess the novel rare diketopiperazine–isoindolinone hybrid skeleton and are discovered in nature for the first time, adding new structural types to fungal diketopiperazine natural products. New compound **4** is structurally similar to the known compound **5**, representing a carboxylated derivative. Notably, bioactivity screening revealed that **4** and **5** both exhibited strong antioxidant activity (IC_50_ 6.01 and 7.00 μg·mL^−1^, respectively), significantly better than the other compounds. This suggests that the introduction of a carboxyl group may enhance radical scavenging ability, providing a reference for structure-based antioxidant design. Although compounds **5**–**7** are known structures, their isolation from *A. nidulans* is reported here for the first time, indicating untapped metabolic potential in this strain.

From a methodological perspective, the use of heterologous expression as a trigger of “metabolic perturbation” to activate cryptic BGCs provides a novel strategy for microbial natural product discovery. However, this strategy currently faces notable challenges, including the unpredictability of activation efficiency and the lack of a clear mechanistic understanding. Further investigations should integrate multi-omics analyses (transcriptomics, metabolomics, and epigenomics) to systematically dissect the global alterations in regulatory networks induced by heterologous expression while rationally designing expression constructs or co-expressing key regulatory factors to enhance the efficiency and specificity of cryptic BGCs activation.

In summary, this study not only reports several structurally novel and biologically active natural products but also reveals a new phenomenon of activating silent gene clusters through heterologous expression perturbation of the host metabolic network, providing new insights and experimental basis for fungal natural product discovery and development of synthetic biology chassis cells.

## Figures and Tables

**Table 1:** NMR spectroscopic data of compounds **1**–**3** (700 MHz for ^1^H NMR and 175 MHz for ^13^C NMR in DMSO-*d_6_*).

No.	1		2		3	
	*δ*_C_, Type	*δ*_H_, Multi. (*J* in Hz)	*δ*_C_, Type	*δ*_H_, Multi. (*J* in Hz)	*δ*_C_, Type	*δ*_H_, Multi. (*J* in Hz)
1		8.12, s, 1-NH		8.05, d (2.4), 1-NH		8.11, s, 1-NH
2	167.9, C		167.0, C		167.0, C	
3	53.9, CH	3.78, td (7.1, 3.8)	55.3, CH	4.17, ddt (4.8, 3.6, 2.0)	58.8, CH	3.72, dt (3.6, 1.8)
4		8.12, s, 4-NH		8.12, d (2.4), 4-NH		7.97, s, 4-NH
5	167.9, C		166.2, C		167.9, C	
6	53.9, CH	3.78, td (7.1, 3.8)	53.7, CH	3.58, ddd (6.8, 3.7, 2.1)	53.7, CH	3.82, m
7	32.8, CH_2_	1.69, m	33.0, CH_2_	1.11, m 0.71, m	33.0, CH_2_	1.67, ddt (13.3, 10.1, 6.0)
8	21.8, CH_2_	1.30, m	21.0, CH_2_	0.71, m	21.9, CH_2_	1.31, m
9	27.6, CH_2_	1.58, p (7.5)	27.4, CH_2_	1.30, m	27.6, CH_2_	1.59, ddd (12.5, 8.9, 6.4)
10	41.5, CH_2_	3.45, td (7.0, 2.0)	41.5, CH_2_	3.27, td (7.1, 3.4)	41.6, CH_2_	3.45, t (7.2)
11	167.1, C		167.0, C		167.0, C	
13	47.9, CH_2_	4.52, s	48.0, CH_2_	4.46, s	48.0, CH_2_	4.51, s
14	153.3, C		153.4, C		153.4, C	
15	118.9, C		118.9, C		118.9, C	
16	156.5, C		156.5, C		156.5, C	
17	103.0, CH	6.82, s	103.0, CH	6.83, s	103.4, CH	6.82, s
18	120.7, C		120.7, C		120.7, C	
19	131.9, C		132.0, C		132.0, C	
20	58.8, CH_3_	3.87, s	58.9, CH_3_	3.87, s	58.9, CH_3_	3.87, s
21	9.4, CH_3_	2.05, s	9.4, CH_3_	2.06, s	9.4, CH_3_	2.06, s
22			38.2, CH_2_	3.13, dd (13.5, 3.9) 2.84, dd (13.5, 5.0)	38.0, CH	1.83, ddp (16.7, 7.1, 3.5)
23			136.2, C		24.3, CH_2_	1.39, dqd (14.0, 7.0, 6.5, 3.8)
24			130.4, CH	7.14, m	15.1, CH_3_	0.89, d (7.1)
25			128.0, CH	7.22, m	11.9, CH_3_	0.83, t (7.4)
26			126.7, CH	7.22, m		
16-OH		9.77, s		9.78, s		9.77, s

**Table 2:** NMR spectroscopic data of compound **4** (500 MHz for ^1^H NMR and 125 MHz for ^13^C NMR in CD_3_OD).

No.	4	
	*δ*_C_, Type	*δ*_H_, Multi. (*J* in Hz)
1	80.7, CH	5.01, ddd (10.5, 6.7, 3.7)
2	28.5, CH_2_	3.18, dd (16.7, 3.8) 2.78, dd (16.8, 10.5)
3	125.6, C	
4	100.2, C	
5	159.1, C	
6	101.9, CH	6.27, s
7	155.7, C	
8	135.9, C	
9	128.0, CH	5.71, dd (15.3, 6.8)
10	135.1, CH	6.38, dd (15.3, 10.4)
11	131.7, CH	6.11, ddd (15.0, 10.4, 1.8)
12	132.9, CH	5.82, dq (14.0, 6.7)
13	18.3, CH_3_	1.77, dd (6.7, 1.6)
14	171.8, C	

**Table 3:** The DPPH free radical scavenging activities of compounds **1**–**10** (μg·mL^−1^).

Compounds	IC_50_
**1**	>500
**2**	>500
**3**	>500
**4**	6.01
**5**	7.00
**6**	34.99
**7**	44.94
**8**	35.14
**9**	>500
**10**	>500
Vc	3.10

## Data Availability

The data presented in this study are available in this article and the Supporting Information.

## Supplementary Materials

The following supporting information can be downloaded at https://www.mdpi.com/article/10.3390/jof12060401/s1, Table S1: Strains used in the study; Table S2: Plasmids used in the study; Table S3: Primers used in the study; Media and Buffers; Figure S1: ^1^H NMR of compound **1** in DMSO-*d*_6_ (700 MHz); Figure S2: ^13^C NMR of compound **1** in DMSO-*d*_6_ (175 MHz); Figure S3: ^1^H-^1^H COSY of compound **1** in DMSO-*d*_6_; Figure S4: HSQC of compound **1** in DMSO-*d*_6_; Figure S5: HMBC of compound **1** in DMSO-*d*_6_; Figure S6: ^1^H NMR of compound **2** in DMSO-*d*_6_ (700 MHz); Figure S7: ^13^C NMR of compound **2** in DMSO-*d*_6_ (175 MHz); Figure S8: ^1^H-^1^H COSY of compound **2** in DMSO-*d*_6_; Figure S9: HSQC of compound **2** in DMSO-*d*_6_; Figure S10: HMBC of compound **2** in DMSO-*d*_6_; Figure S11: NOESY of compound **2** in DMSO-*d*_6_; Figure S12: ^1^H NMR of compound **3** in DMSO-*d*_6_ (700 MHz); Figure S13: ^13^C NMR of compound **3** in DMSO-*d*_6_ (175 MHz); Figure S14: ^1^H-^1^H COSY of compound **3** in DMSO-*d*_6_; Figure S15: HSQC of compound **3** in DMSO-*d*_6_; Figure S16: HMBC of compound **3** in DMSO-*d*_6_; Figure S17: ^1^H NMR of compound **4** in CD_3_OD (700 MHz); Figure S18: ^13^C NMR of compound **4** in CD_3_OD (175 MHz); Figure S19: ^1^H-^1^H COSY of compound **4** in CD_3_OD; Figure S20: HSQC of compound **4** in CD_3_OD; Figure S21: HMBC of compound **4** in CD_3_OD; Figure S22: Infrared (IR) spectrum of compound **4**; Figure S23: RT-PCR assays of partial genes in the recombinant strain AN-*tcaABCDEFGHI*.
