# Supplementary material for: Heterologous Expression Unexpectedly Activates the Host Cryptic Genes in Aspergillus nidulans and Enables the Discovery of Novel Natural Products
Source: J Fungi (Basel). 2026 Jun 1;12(6):401. doi: 10.3390/jof12060401 (PMC13302354; doi:10.3390/jof12060401)
Supplement: Supplementary file 1 [file jof-12-00401-s001.zip › jof-4324355-supplementary -bai2.pdf]

Supporting Information

Heterologous Expression Unexpectedly Activates the Host Cryptic Genes in *Aspergillus nidulans* and Enables the Discovery of Novel Natural Products

Cong Liu [†], Yinan Hao [†], Siyuan Qi and Jian Bai *

State Key Laboratory of Bioactive Substance and Function of Natural Medicines, Institute of Materia Medica, Chinese Academy of Medical Sciences & Peking Union Medical College, Beijing 100050, China

* Correspondence: baijian@imm.ac.cn (J.B.)

† These authors contributed equally to this work.

Table of Contents

Table S1. Strains used in the study. **S2**

Table S2. Plasmids used in the study. **S2**

Table S3. Primers used in the study. **S3**

Media and Buffers **S4**

Spectroscopic Data for New Compounds

Figure S1-S5. NMR spectra of compound **1** **S5–S9**

Figure S6-S11. NMR spectra of compound **2** **S10–S15**

Figure S12-S16. NMR spectra of compound **3** **S16–S20**

Figure S17-S22. NMR and IR spectra of compound **4** **S21–S26**

Structure Elucidation of Known Compounds **S27–S28**

Method and Results of Reverse Transcription PCR (RT-PCR) **S29–S30**

Figure S23. RT-PCR assays of partial genes in the recombinant strain AN-*tcaABCDEFGHI* **S30**

Reference **S31**

Table S1. Strains used in the study.

Strains	Description
Penicillium dangeardii	Wild type strain[1]
Calcarisporium arbuscula	Wild type strain[2]
Escherichia coli XL1-Blue	*recA1 endA1 gyrA96 thi-1 hsdR17supE44 relA1 lac* [*F' pro AB lac IqZΔM15 Tn10 (Tetr)*]
Aspergillus nidulans A8030::empty vectors	*A. nidulans* A8030 harboring pANP, pANR, and pANU
Saccharomyces cerevisiae BJ5464-NpgA	Host for plasmid assembly via homologous recombination
A. nidulans::*nrpACDEFGHI*	*A. nidulans* harboring plasmid pANP-*nrp*1, pANR-*nrp*2, pANU-*nrp*3.
A. nidulans::*nrpEFGHI*	*A. nidulans* harboring plasmid pANP-*nrp*1, pANU-*nrp*3.
A. nidulans::*tcaABCDEFGHI*	*A. nidulans* harboring plasmid pANP-*tca*1, pANR-*tca*2, pANU-*tca*3.

Table S2. Plasmids used in the study.

Plasmids	Description
pANP-*nrp*1	pANP-*nrp*1 carrying *nrpG* under *AmyB* promoter.
pANR-*nrp*2	pANR-*nrp*2 carrying *nrpA*, *nrpC*, *nrpD* driven by *gpdA*, *AmyB* and *glaA* promoter, respectively.
pANU-*nrp*3	pANU-*nrp*3 carrying *nrpE*, *nrpF*, *nrpH*, *nrpI* driven by *gpdA*, *AmyB*, *glaA* and *Ptub* promoter, respectively.
pANP-*tca*1	pANP-*tca*1 carrying *tcaC, tcaD, tcaE* driven by *gpdA*, *AmyB* and *glaA* promoter, respectively.
pANR-*tca*2	pANR-*tca*2 carrying *tcaA*, *tcaF*, *tcaG* driven by *gpdA*, *AmyB* and *glaA* promoter, respectively.
pANU-*tca*3	pANU-*tca*3 carrying *tcaB*, *tcaH*, *tcaI* driven by *gpdA*, *AmyB* and *glaA* promoter, respectively.

Table S3. Primers used in the study.

Name	Sequence (5′→3′)
nrpA-F	cttcatccccagcatcattacacctcagcaATGTCTCAAATACGACTCGTGTGCA
nrpA-R	tcatttatagctcgttcggcacctttaatcAGTGGTATCACCAGAAATCCCAGT
nrpC-F	tctgaacaataaaccccacagaaggcatttATGCGCGTTGAACCTACATCCA
nrpC-R	agcgctcaccaagctcttaaaacgggaattATTTTCGGAGGCTCGGTTTGG
nrpD-F	attaccccgccacatagacacatctaaacaATGGAGTCAAAACTGCCACAAGAG
nrpD-R	cgccaggtacgaccagttcggaagatcaggTCAATCCACATACGATTCCTTGTT
nrpE-F	attaccccgccacatagacacatctaaacaATGGCGGATATCAGTGACCCTG
nrpE-R	ccacagtccgtaacactccaacgtctccaaCAAATCGGGCCTTGGAGGGG
nrpF-F	cttcatccccagcatcattacacctcagcaATGGCTTCAGAAAATCGTATCAAG
nrpF-R	tcatttatagctcgttcggcacctttaatcGGATCGAAGGCCAATCTAGTTGAAG
nrpG-F	tctgaacaataaaccccacagaaggcatttATGGCTGGTACAGCTTTGGCCTTC
nrpG-R	agcgctcaccaagctcttaaaacgggaattAAATACTTCCACCAGTCTAATTGAC
nrpH-F	aatcctctatacccgtccacacccttcacaATGGGCAGCCTACCTAGGTATG
nrpH-R	agcgctcaccaagctcttaaaacgggaattCTCTTTCGTGACATCGAAACTGC
nrpI-F	tctgaacaataaaccccacagaaggcatttATGGAAAACATCCAGGTGACACAA
nrpI-R	ctcccgtcacccaaatcaattcaccggagtACTGGCTCAGTCACAAAACCCTAA
tcaA-F	cttcatccccagcatcattacacctcagcaATGACCAACACCGCCTCC
tcaA-R	tcatttatagctcgttcggcacctttaatcCTGCAGTACTGTATTTCTGCCGA
tcaB-F	cttcatccccagcatcattacacctcagcaATGAGGTTCTCTGTTGTCGTTG
tcaB-R	tcatttatagctcgttcggcacctttaatcATACGACACCGAATCGATACCT
tcaC-F	attaccccgccacatagacacatctaaacaATGCAGGAAAAGCTCAACCG
tcaC-R	cgccaggtacgaccagttcggaagatcaggGCAGACTTGGCGGTTTGG
tcaD-F	cttcatccccagcatcattacacctcagcaATGGCGTCCTATGCAGTCG
tcaD-R	agcgctcaccaagctcttaaaacgggaattGCCCAGATAAGTCCCTTTGCG
tcaE-F	tctgaacaataaaccccacagaaggcatttATGGCGCAAGTCGATACCATC
tcaE-R	ctcccgtcacccaaatcaattcaccggagtTGGAACGACAATCGCCGG
tcaF-F	attaccccgccacatagacacatctaaacaATGTCGAAATGTACTAGCAAACGA
tcaF-R	cgccaggtacgaccagttcggaagatcaggATTTGTGATACCCAGCCACAG
tcaG-F	tctgaacaataaaccccacagaaggcatttATGCTGGCCATCCGAGCATTC
tcaG-R	agcgctcaccaagctcttaaaacgggaattCATTGTGCTGACCTTTCTGCCTCT
tcaH-F	tctgaacaataaaccccacagaaggcatttATGGGCCGCAAGCCAAAC
tcaH-R	ctcccgtcacccaaatcaattcaccggagtCAAATCACTTTTGTCATCCATTCGT
tcaI-F	attaccccgccacatagacacatctaaacaATGGGTGCTCTAAAGCTGTTTG
tcaI-R	agcgctcaccaagctcttaaaacgggaattGGCAAATAAATCTGCACGAGTATGT

Media and Buffers

CD (Czapek-Dox) Sporulation Medium: 10.0 g glucose, 50 mL 20×salt solution, 1 mL trace element solution, 7.5 g agar, dissolved in deionized water to 1 L, sterilized by autoclaving at 115 ℃ for 20 min.

CD-ST Fermentation Medium: 20.0 g soluble starch, 20.0 g tryptone, 50 mL 20×salt solution, 1 mL trace element solution, dissolved in deionized water to 1 L, sterilized by autoclaving at 121 ℃ for 20 min.

Osmetic Buffer: 147.7 g $MgSO_4 \cdot 7H_2O$, 14.2 g $Na_2HPO_4 \cdot 12H_2O$, 12.0 g $NaH_2PO_4 \cdot 2H_2O$, dissolved in deionized water, adjusted to pH 5.8 with 1 M Na_2HPO_4, and brought to 500 mL. Filter-sterilized (0.22 μm) .

Trapping Buffer: 0.1 M Tris-HCl, pH 7.0, 109.3 g Sorbitol, dissolved in deionized water to 1 L, sterilized by autoclaving at 115 ℃ for 20 min.

STC Buffer: 218.6 g sorbitol, 1.47 g $CaCl_2 \cdot 2H_2O$, 10 mM Tris-HCl (pH 7.5), dissolved in deionized water to 1 L, sterilized by autoclaving at 115 ℃ for 20 min.

PEG4000 Solution: 60.0 g PEG4000 (BDH), 50 mM $CaCl_2 \cdot 2H_2O$, 50 mM Tris-HCl (pH 7.5), dissolved in deionized water to 100 mL, sterilized by autoclaving at 115 ℃ for 20 min.

Vitamin Supplements: 1000×VB_6 stock (10.0 mg pyridoxine hydrochloride in 100 mL deionized water), 100×VB_2 stock (10.0 mg riboflavin in 100 mL deionized water), 50×uracil/uridine stock (2.8 g uridine and 6.3 g uracil in 100 mL deionized water). All solutions were filter-sterilized (0.22 μm) and stored at 4 ℃ protected from light.

NMR Spectroscopic Data for New Compounds

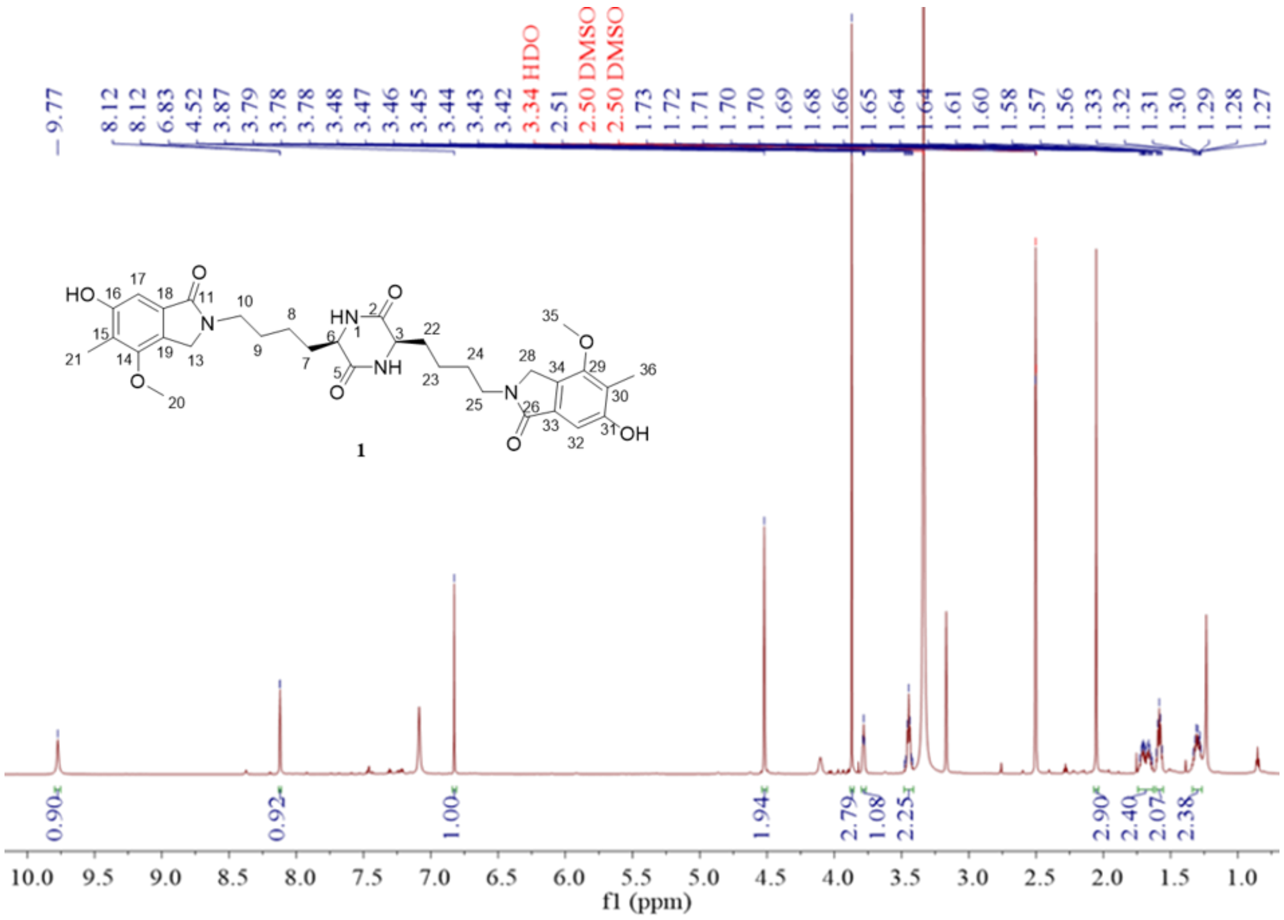

Figure S1. ^{1}H NMR of compound **1** in DMSO-d_6 (700 MHz)

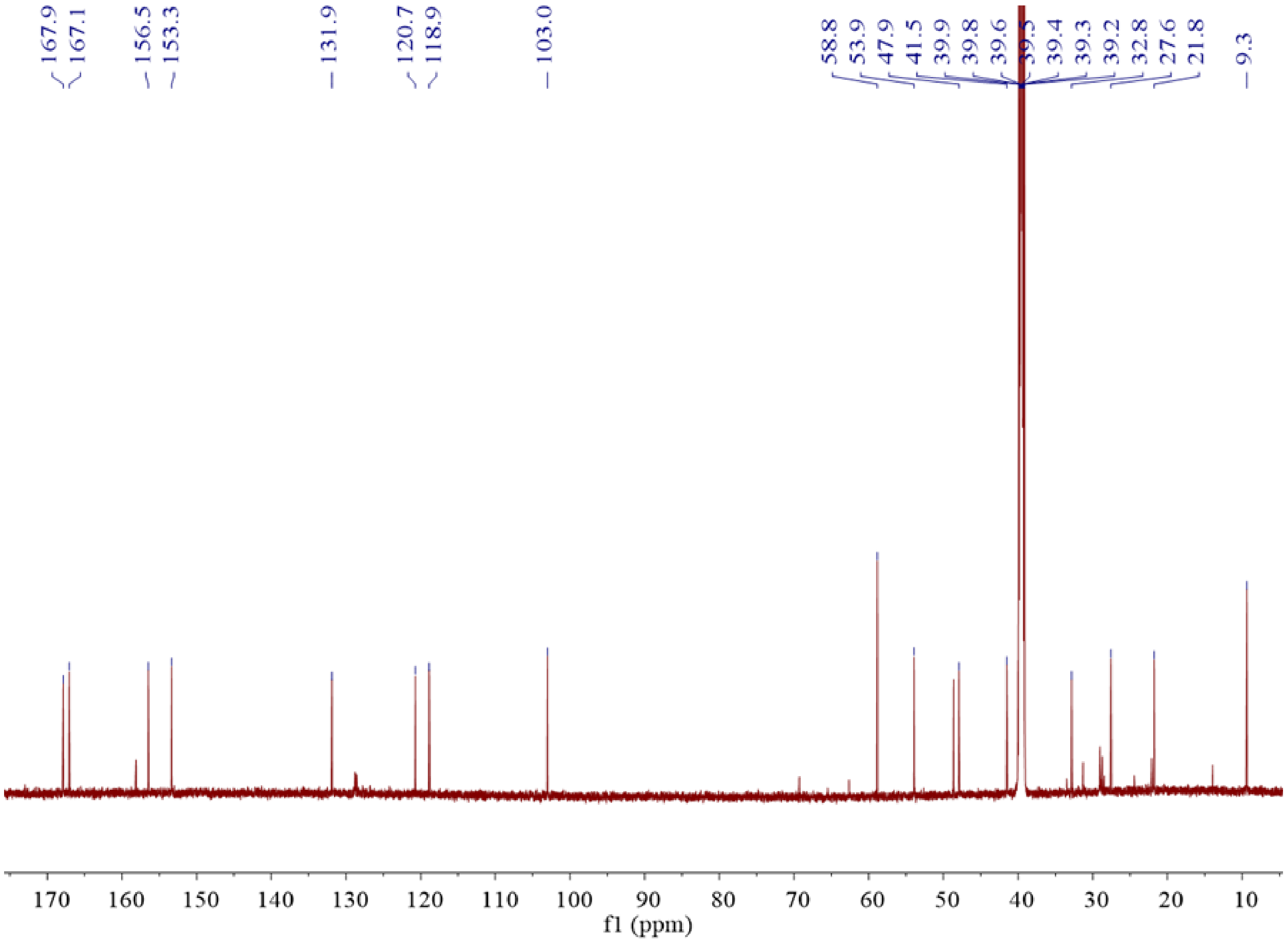

Figure S2. ^{13}C NMR of compound **1** in DMSO-d_6 (175 MHz)

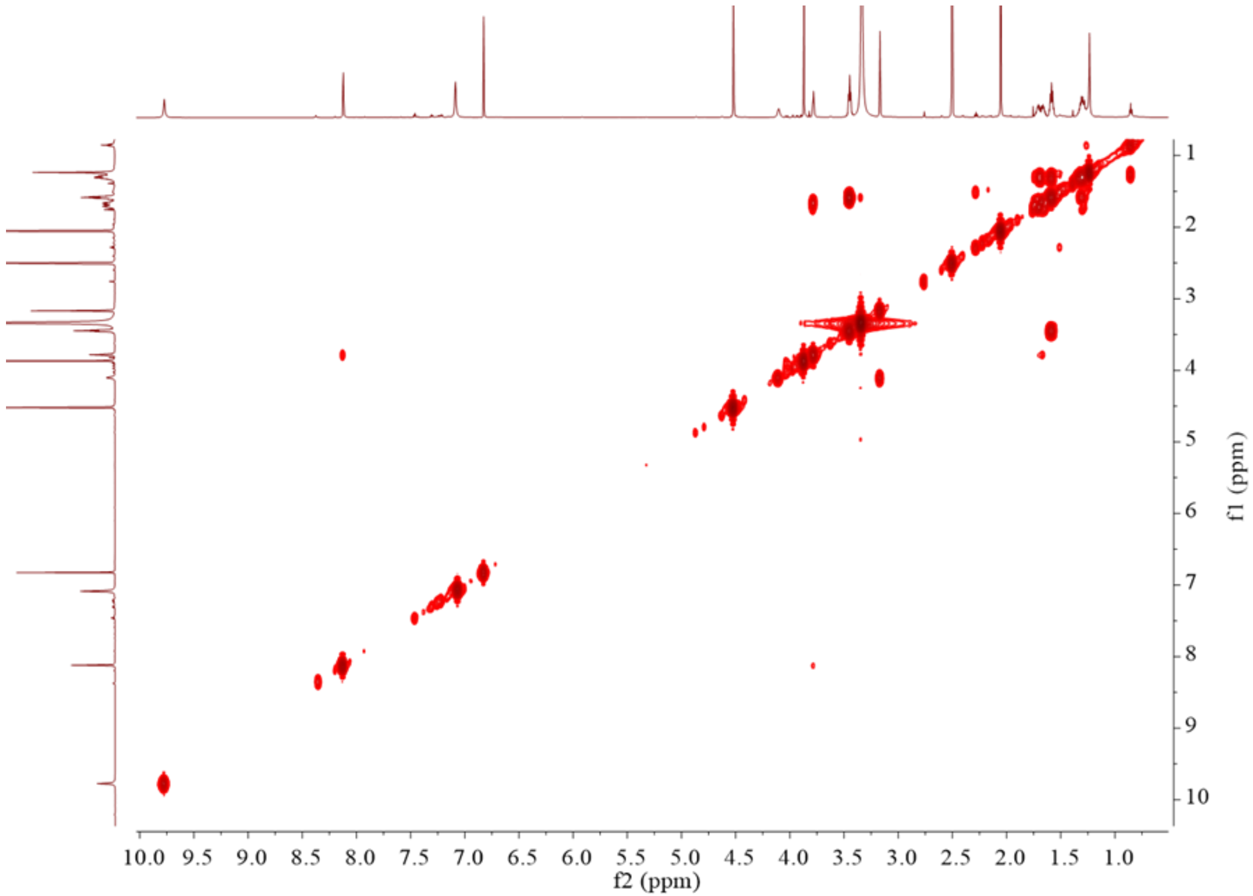

Figure S3. ^{1}H-^{1}H COSY of compound **1** in DMSO-d_6

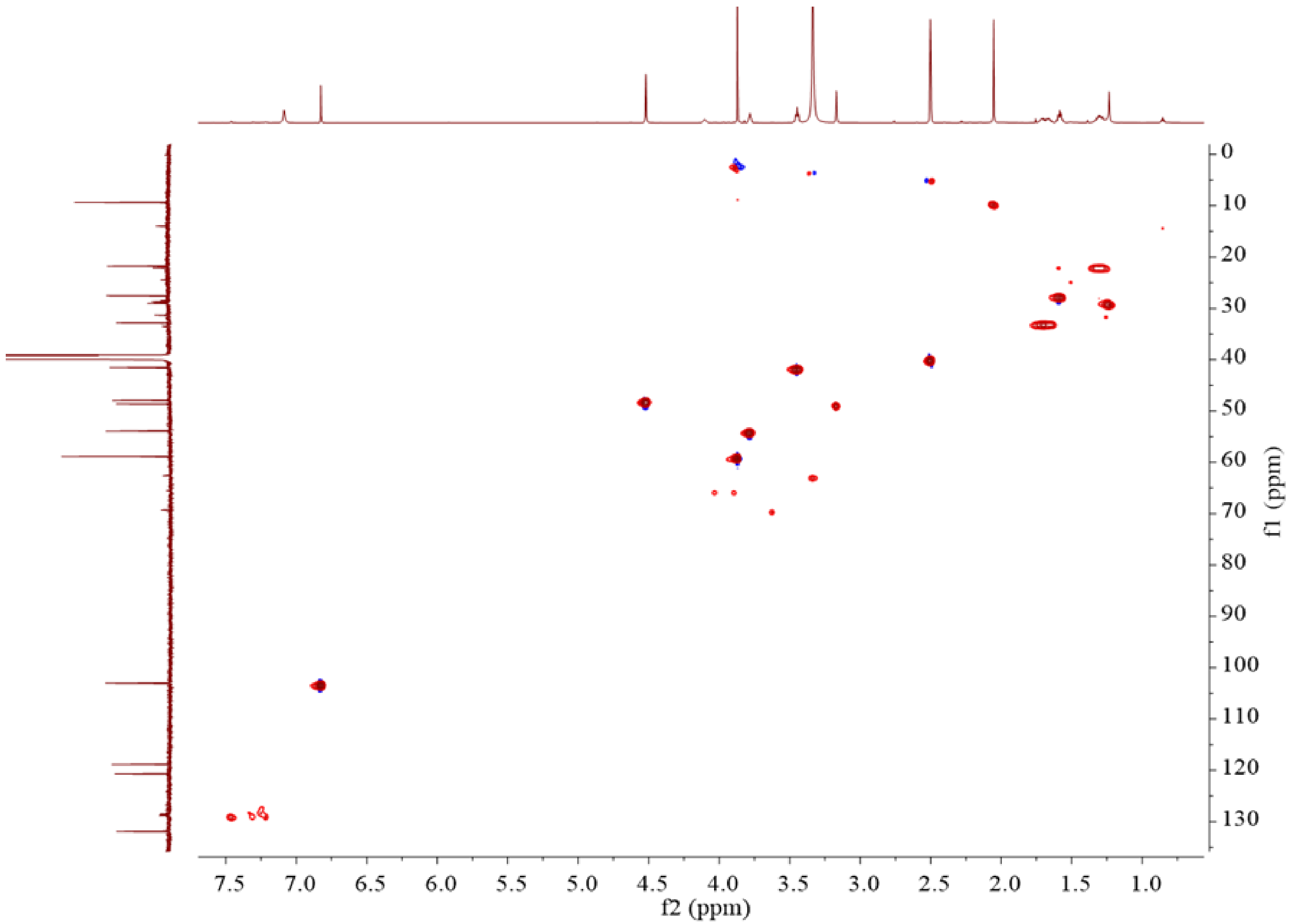

Figure S4. HSQC of compound **1** in DMSO-d_6

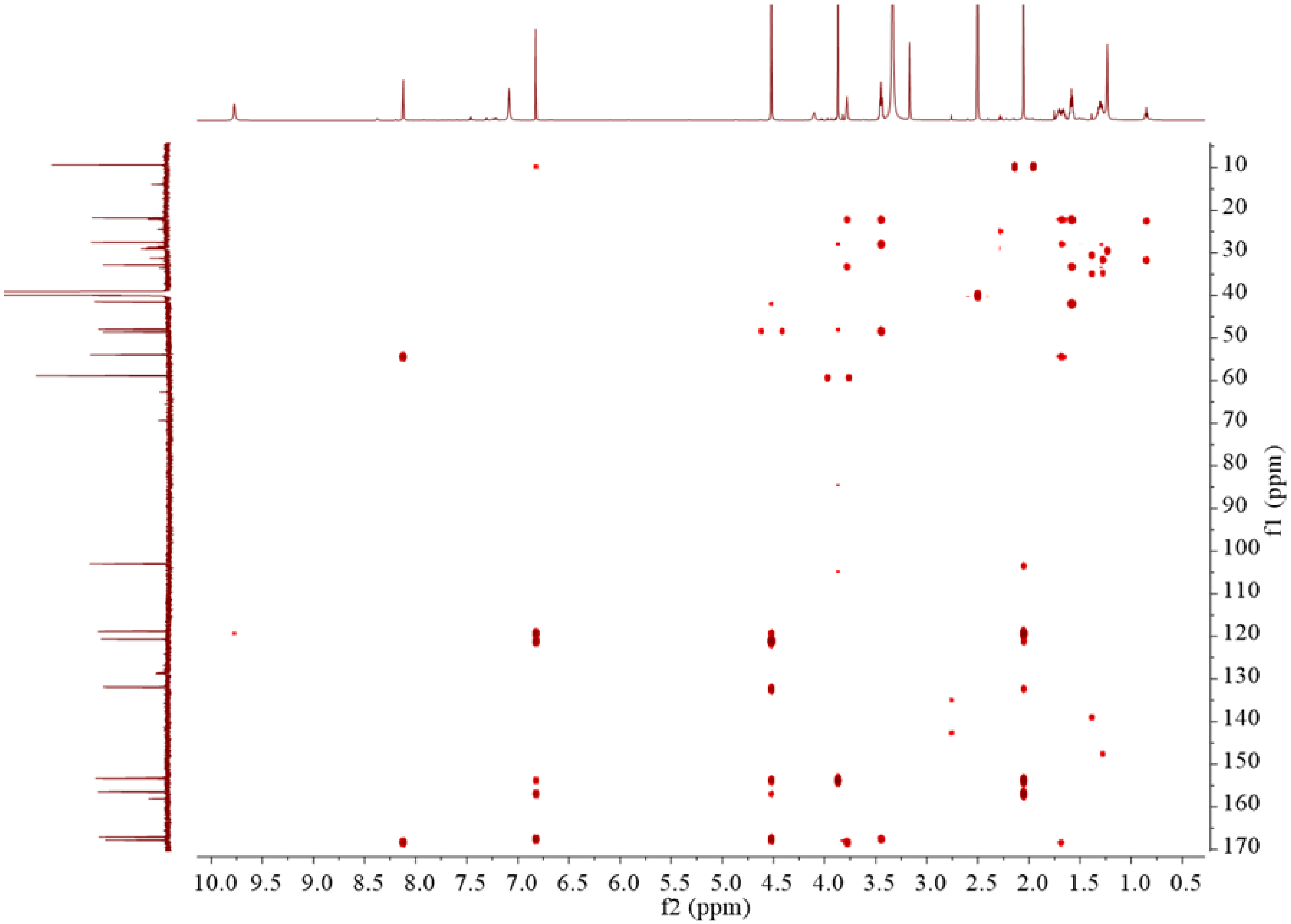

Figure S5. HMBC of compound **1** in DMSO-d_6

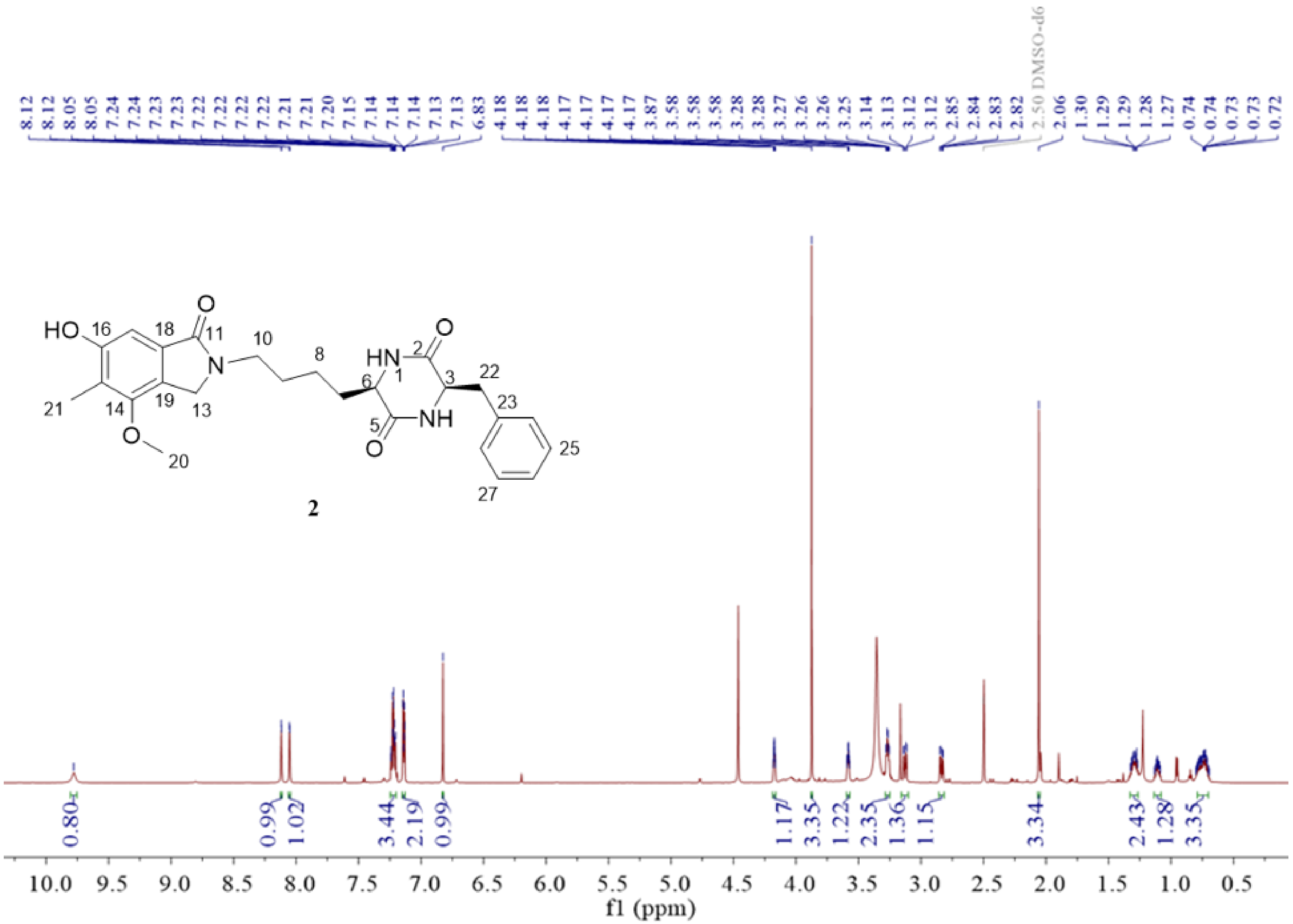

Figure S6. ^{1}H NMR of compound **2** in DMSO-d_6 (700 MHz)

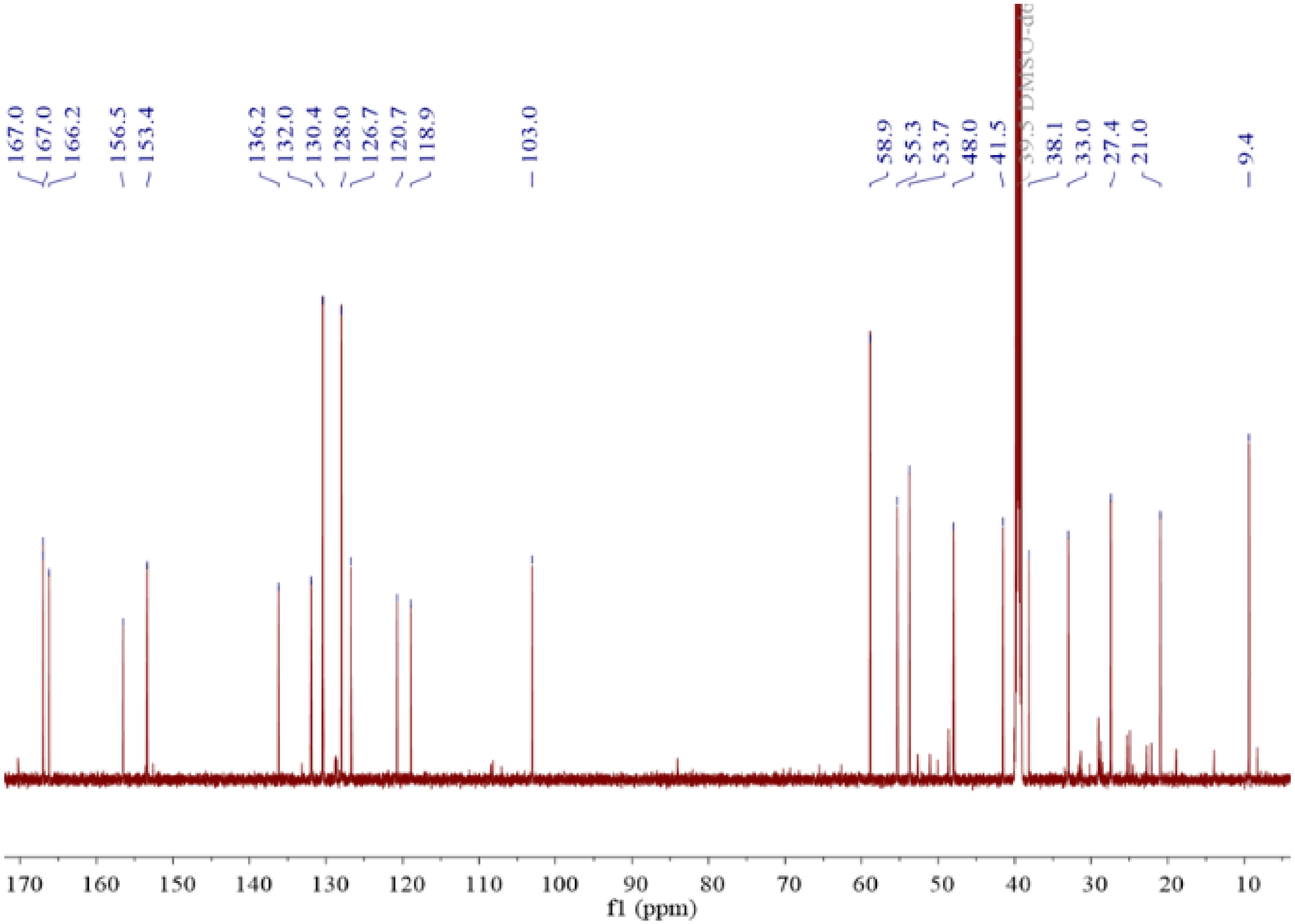

Figure S7. ^{13}C NMR of compound **2** in DMSO-d_6 (175 MHz)

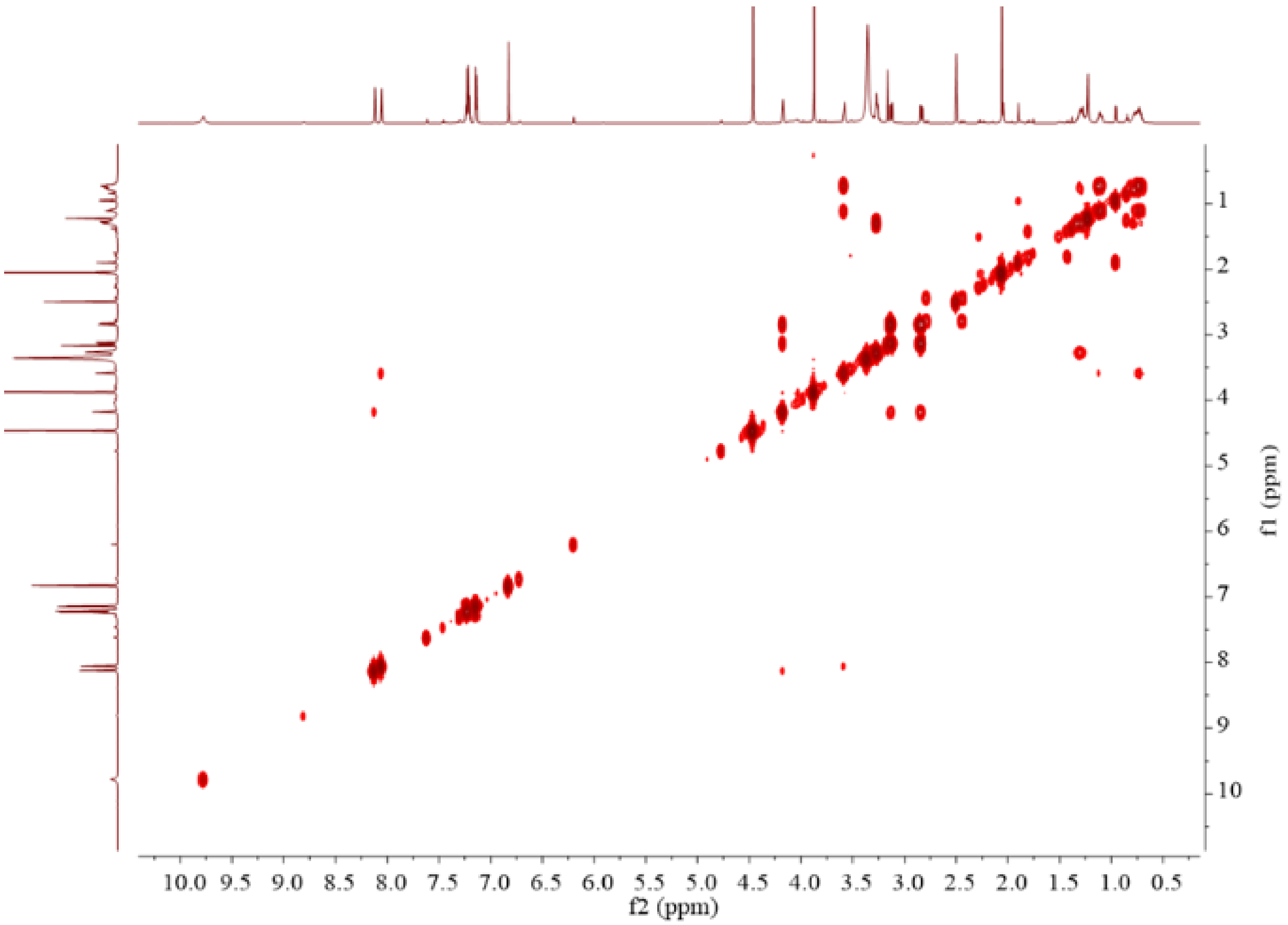

Figure S8. ^{1}H-^{1}H COSY of compound **2** in DMSO-d_6

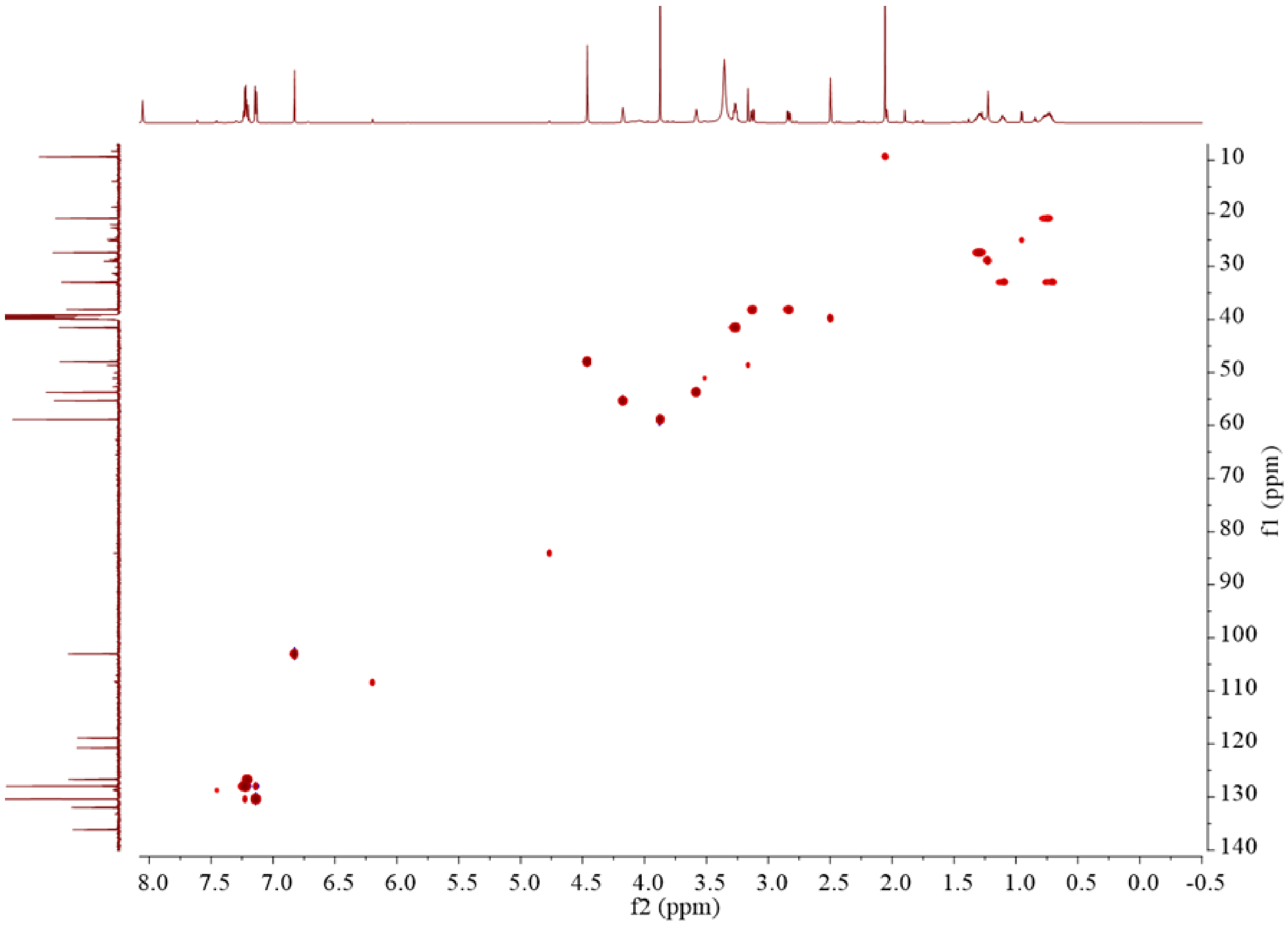

Figure S9. HSQC of compound **2** in DMSO-d_6

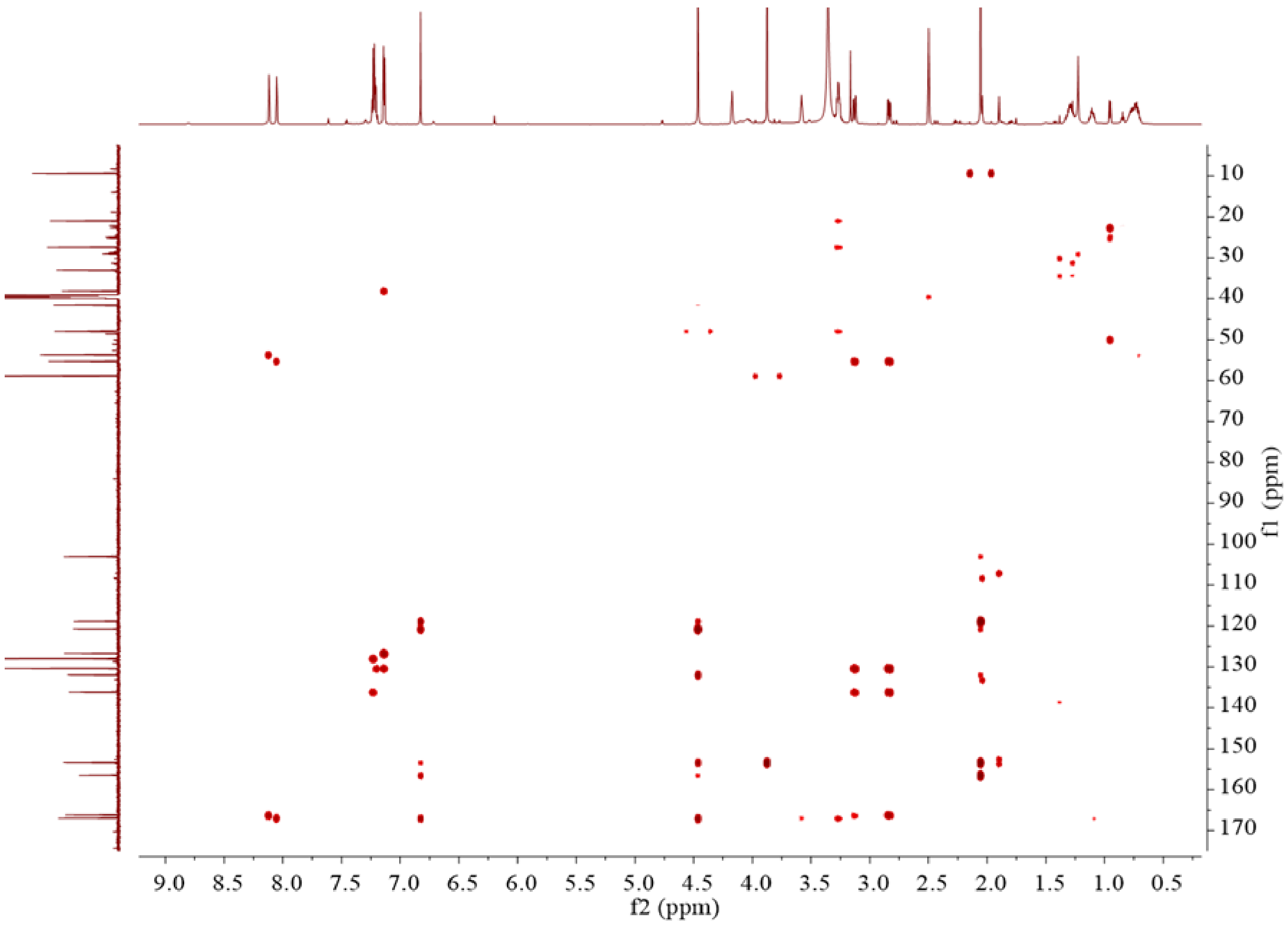

Figure S10. HMBC of compound **2** in DMSO-d_6

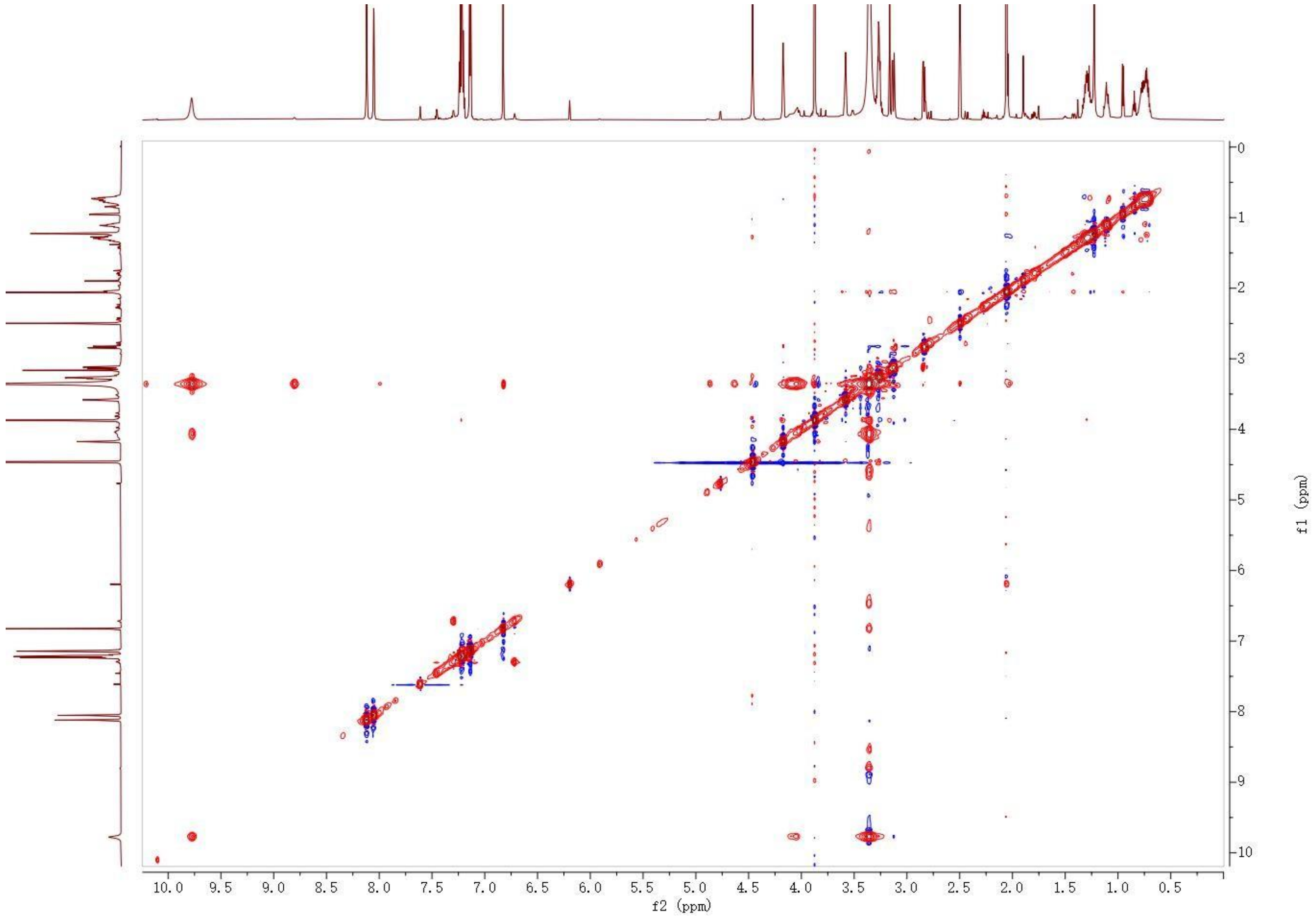

Figure S11. NOESY of compound **2** in DMSO-d_6

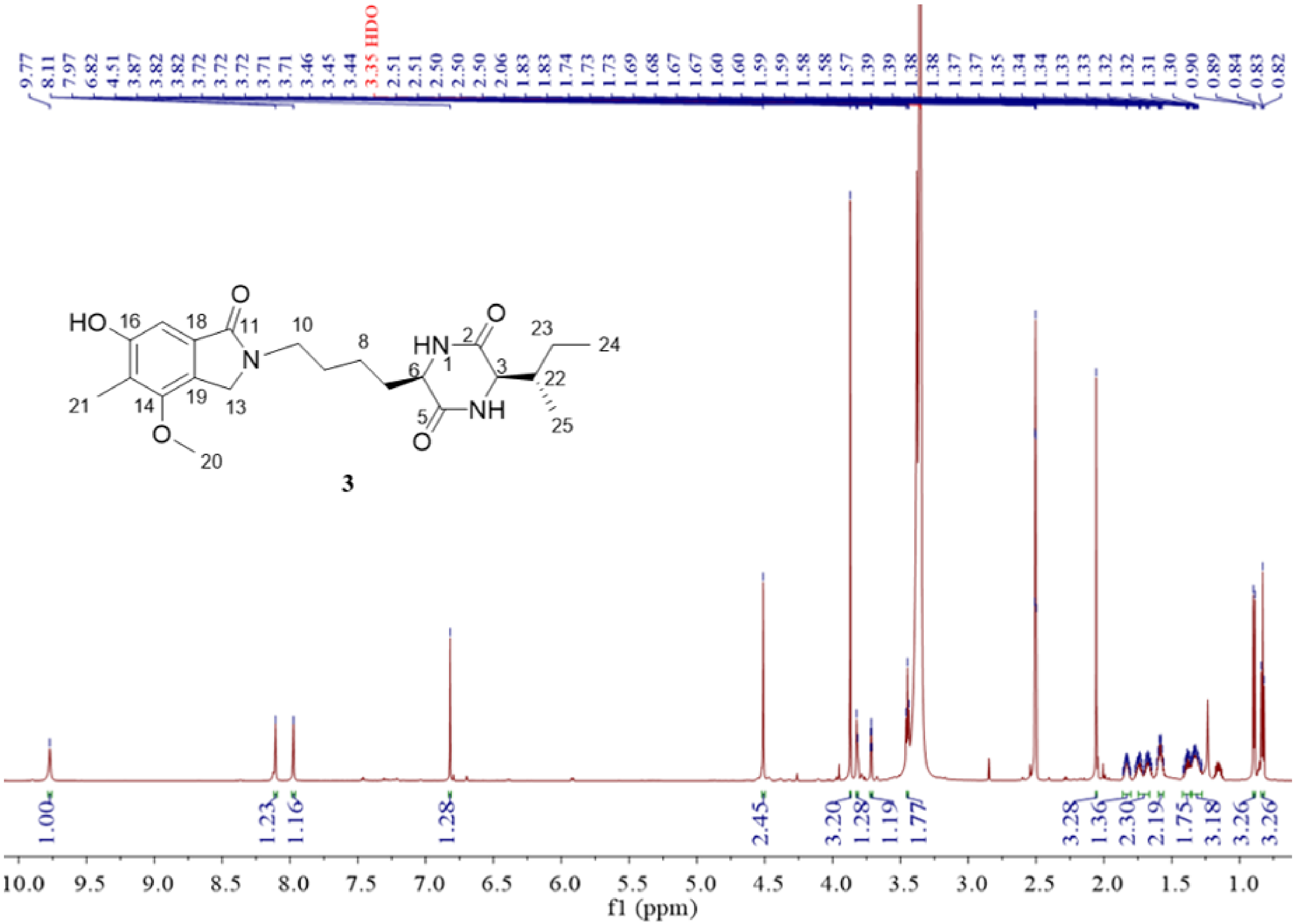

Figure S12. ^{1}H NMR of compound **3** in DMSO-d_6 (700 MHz)

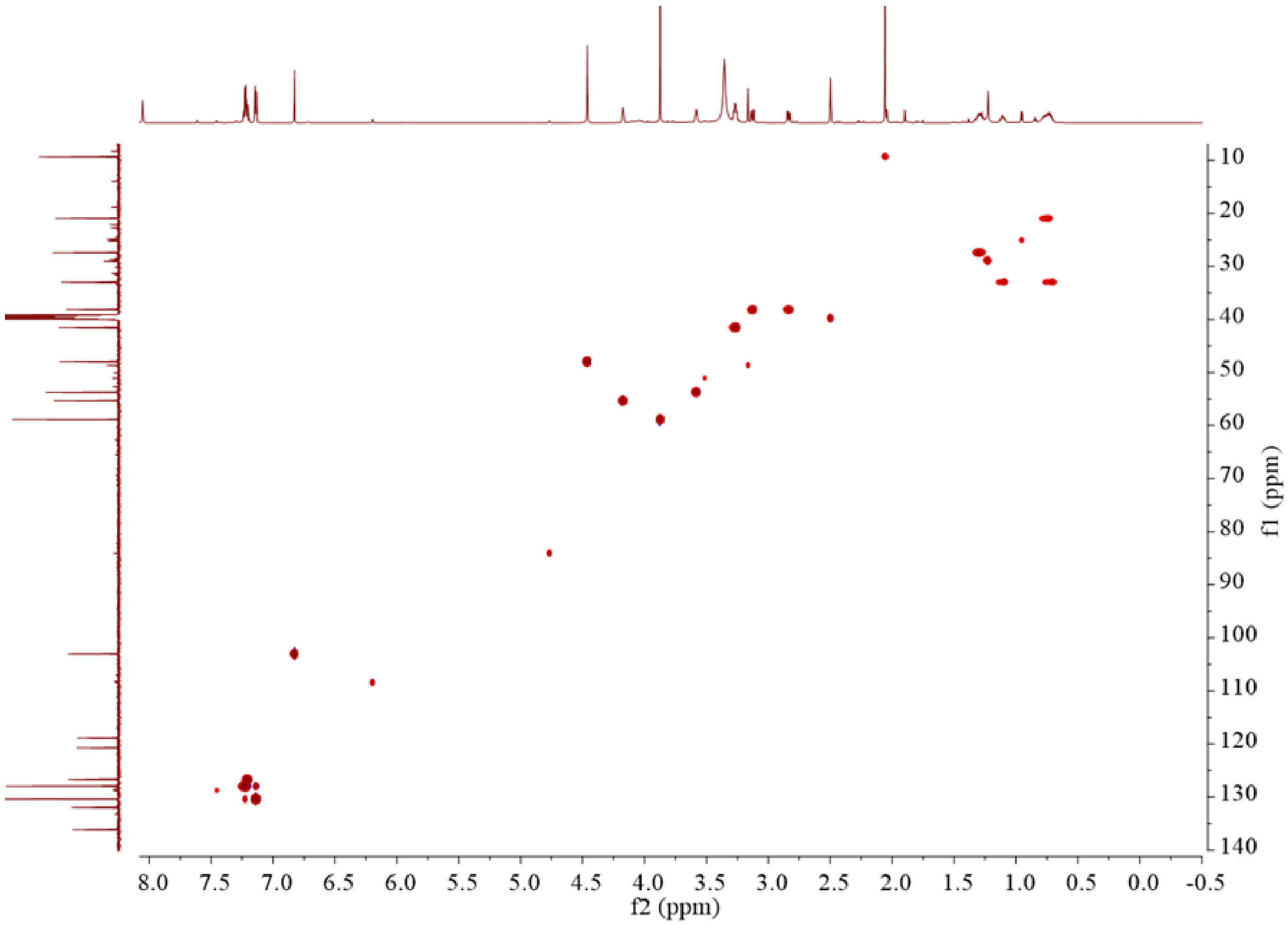

Figure S13. ^{13}C NMR of compound **3** in DMSO-d_6 (175 MHz)

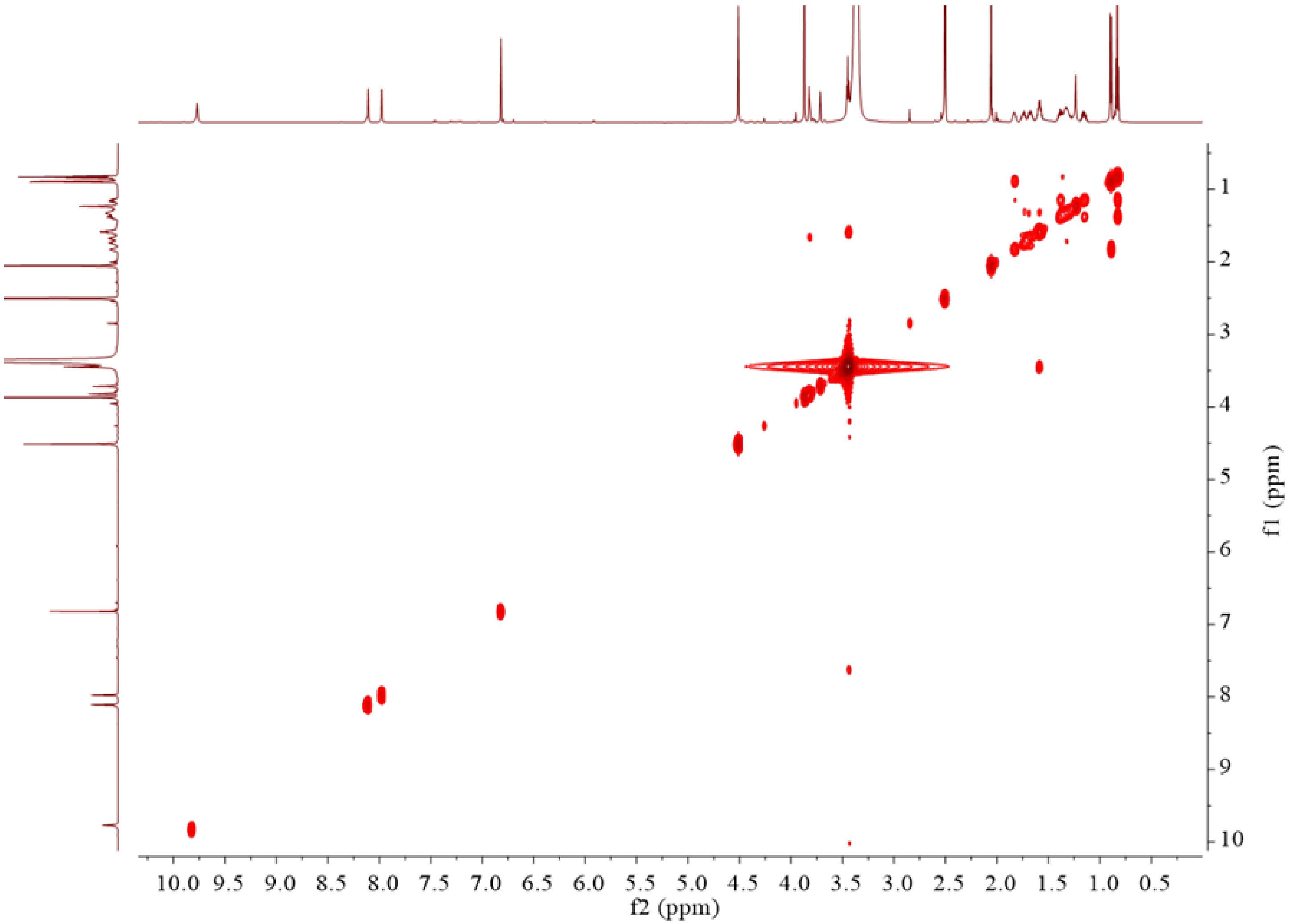

Figure S14. ^{1}H-^{1}H COSY of compound **3** in DMSO-d_6

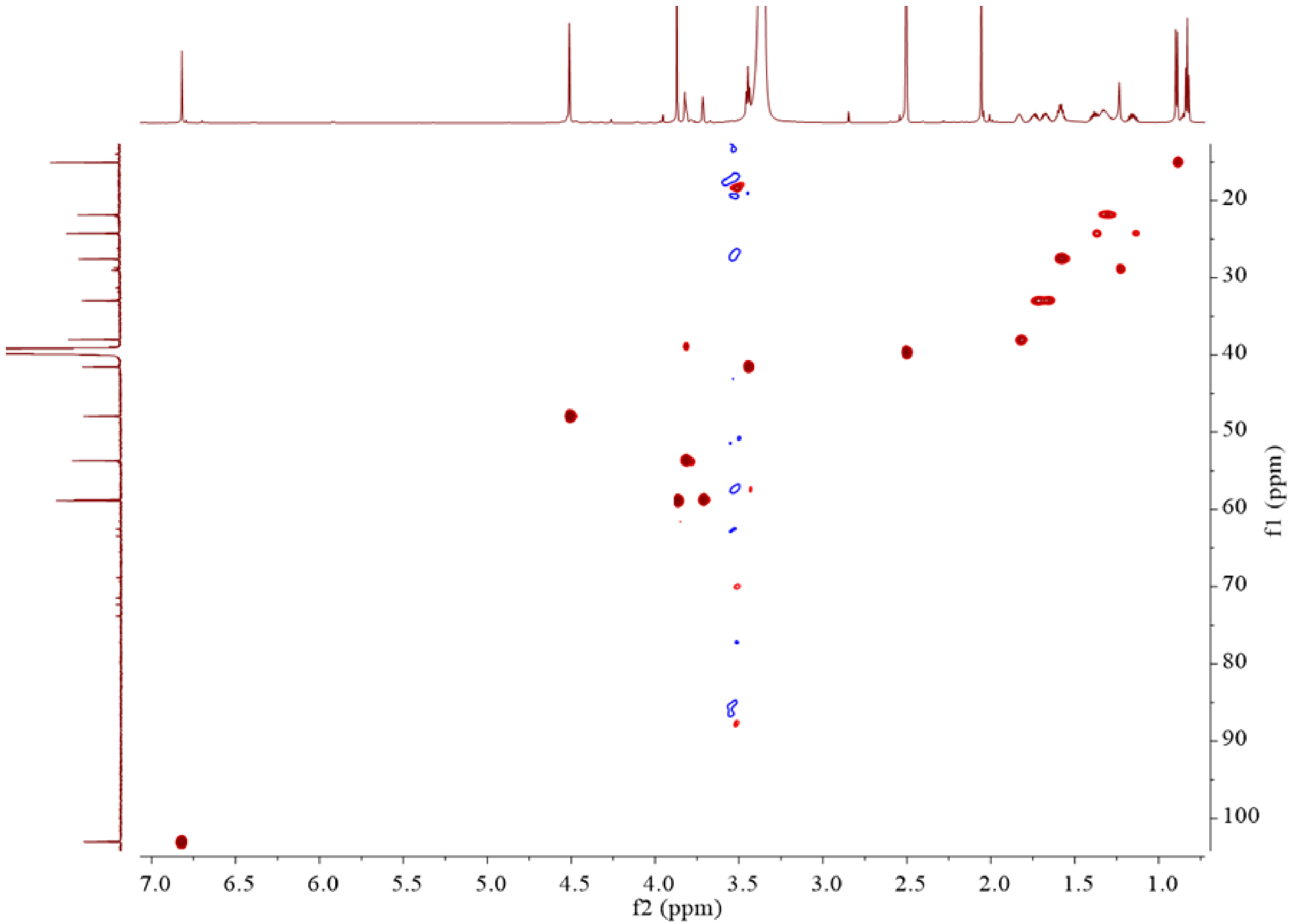

Figure S15. HSQC of compound **3** in DMSO-d_6

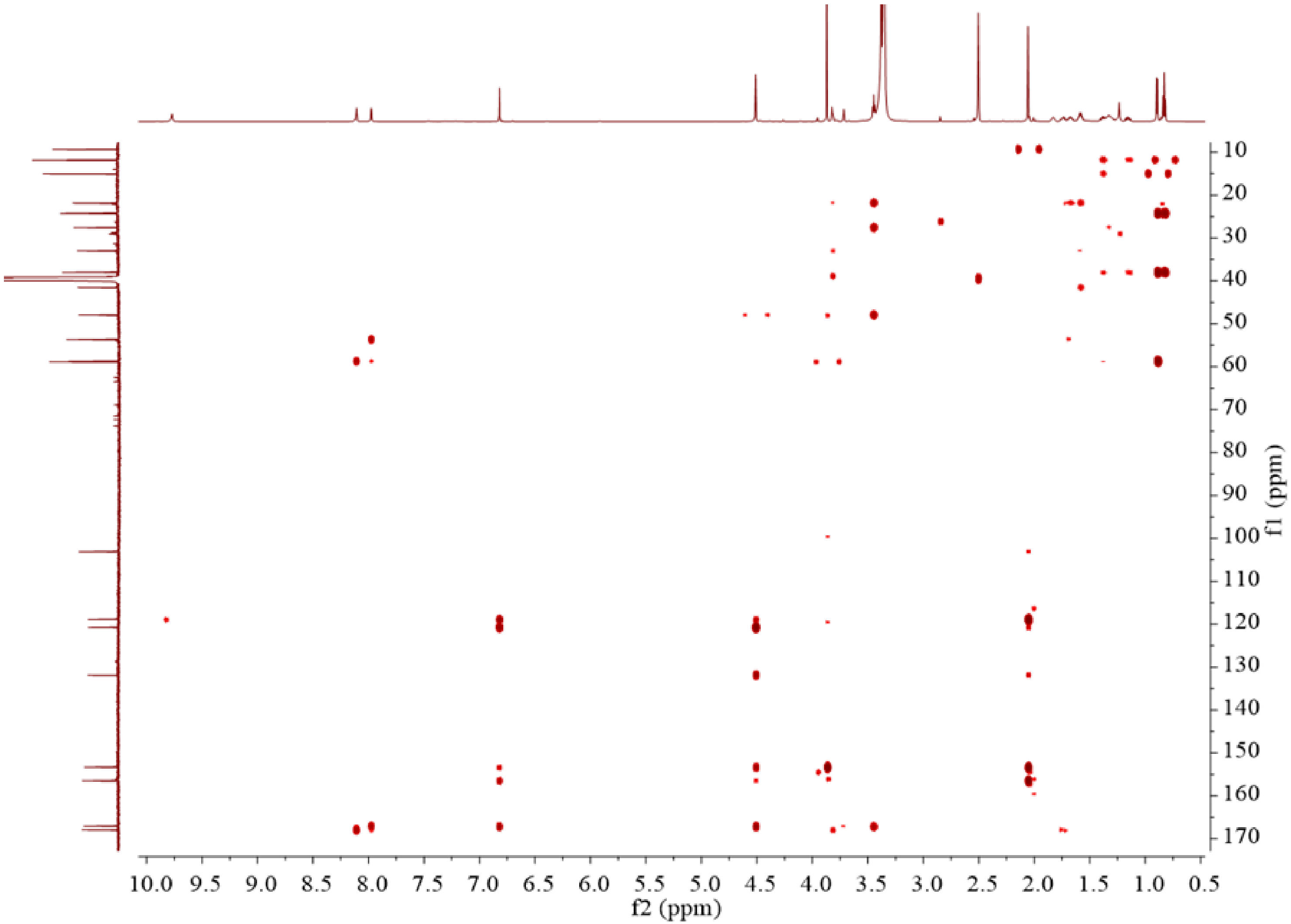

Figure S16. HMBC of compound **3** in DMSO-d_6

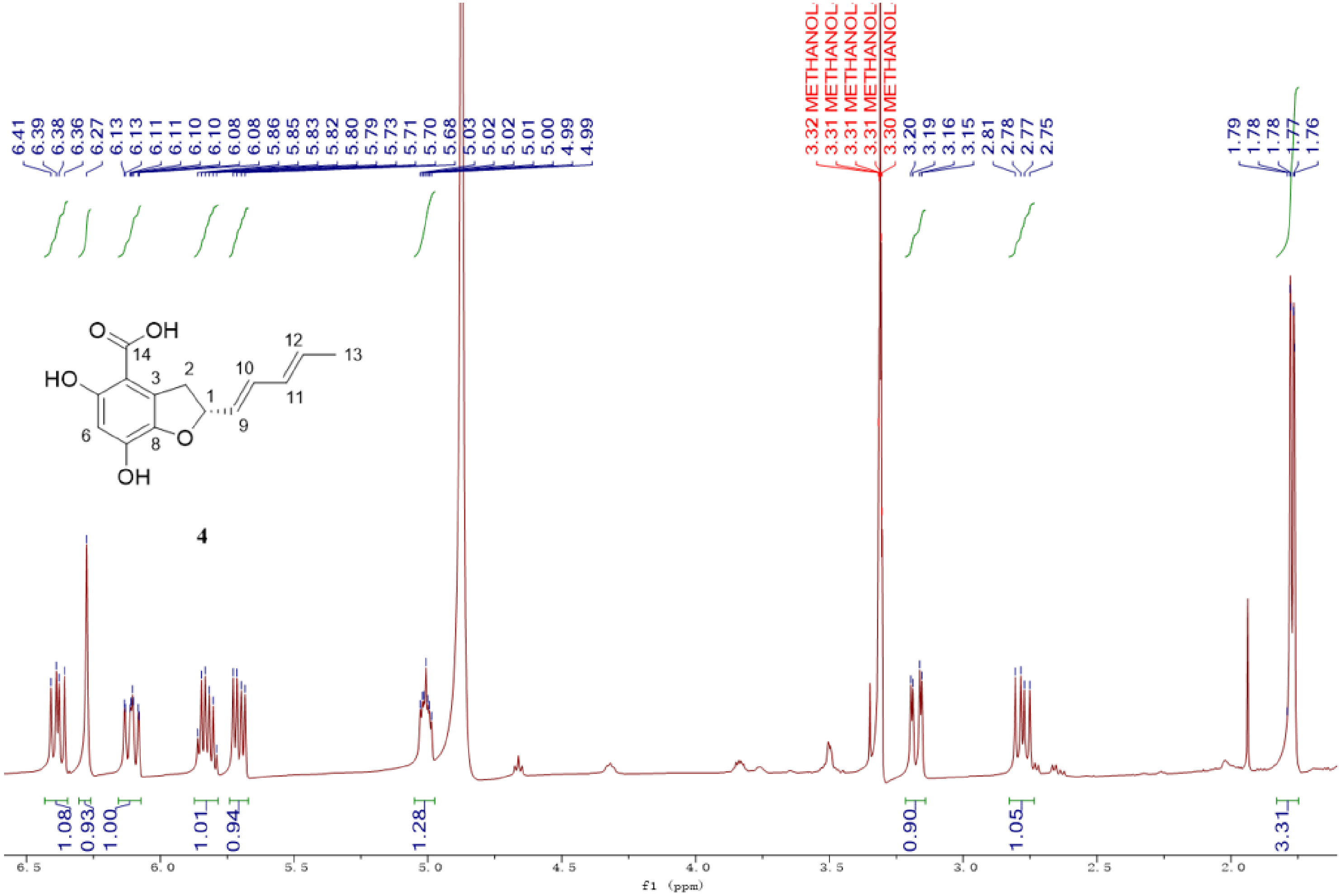

Figure S17. ^{1}H NMR of compound **4** in CD_3OD (700 MHz)

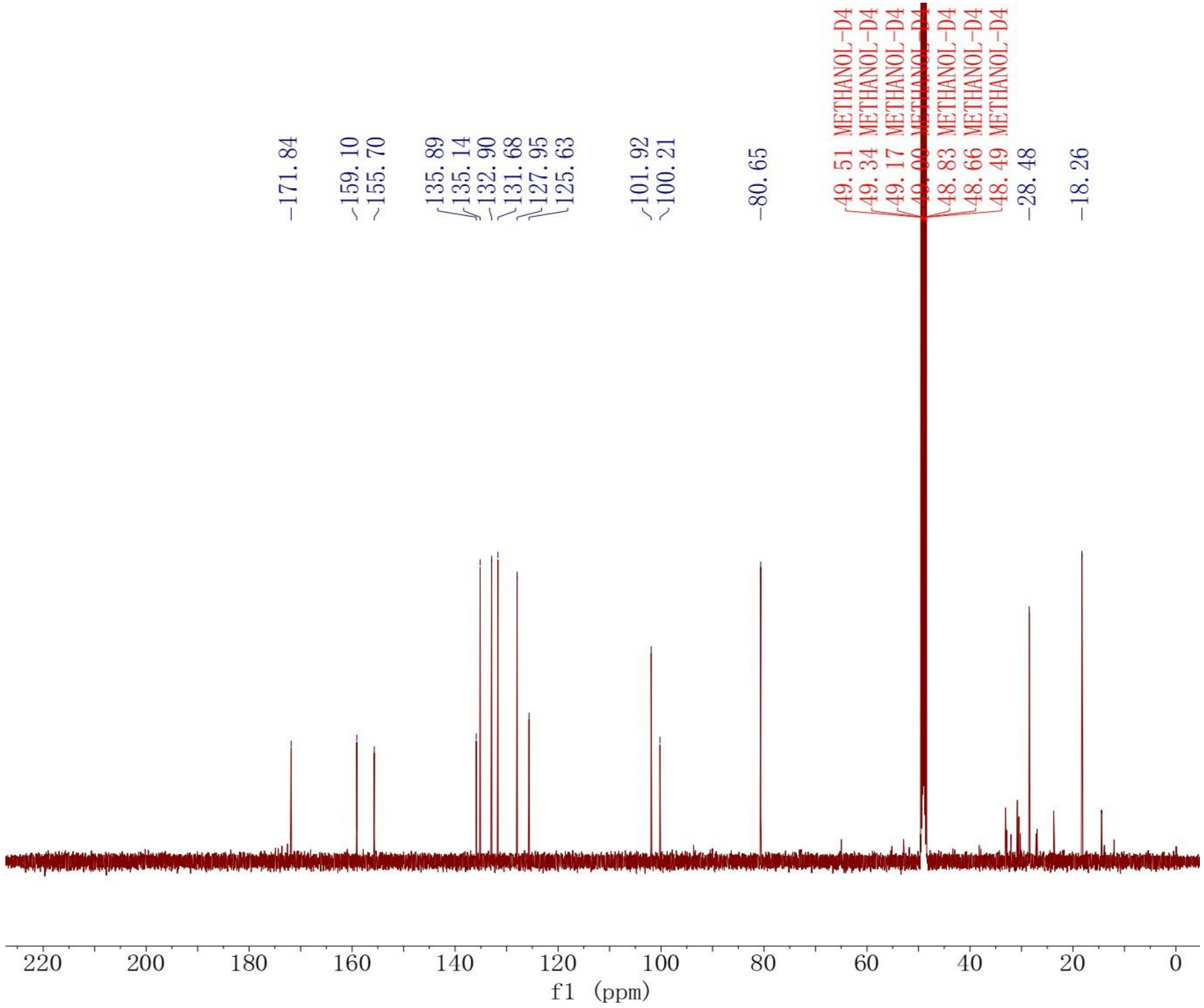

Figure S18. ^{13}C NMR of compound **4** in CD_3OD (175 MHz)

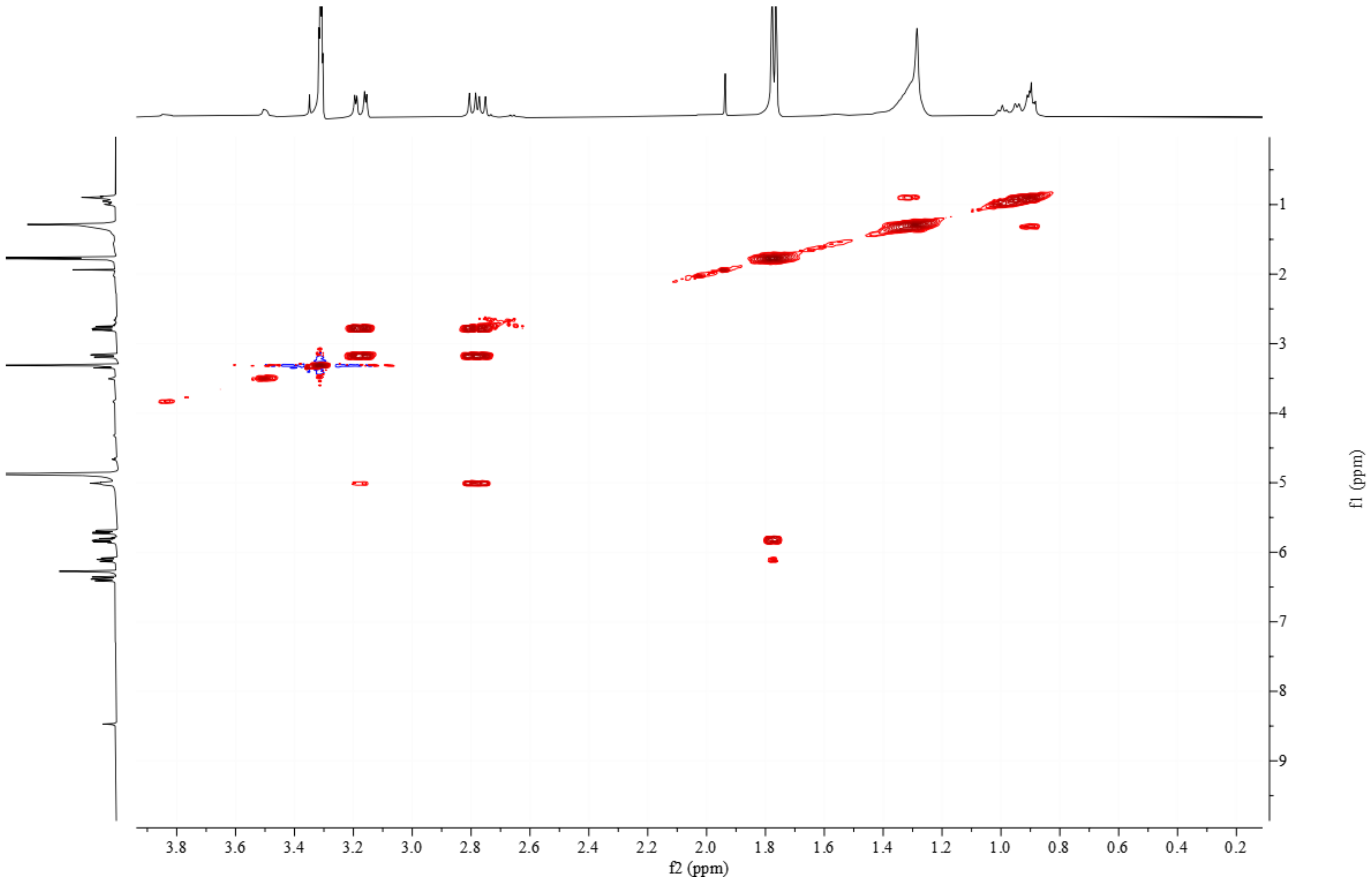

Figure S19. ^{1}H-^{1}H COSY of compound **4** in CD_3OD

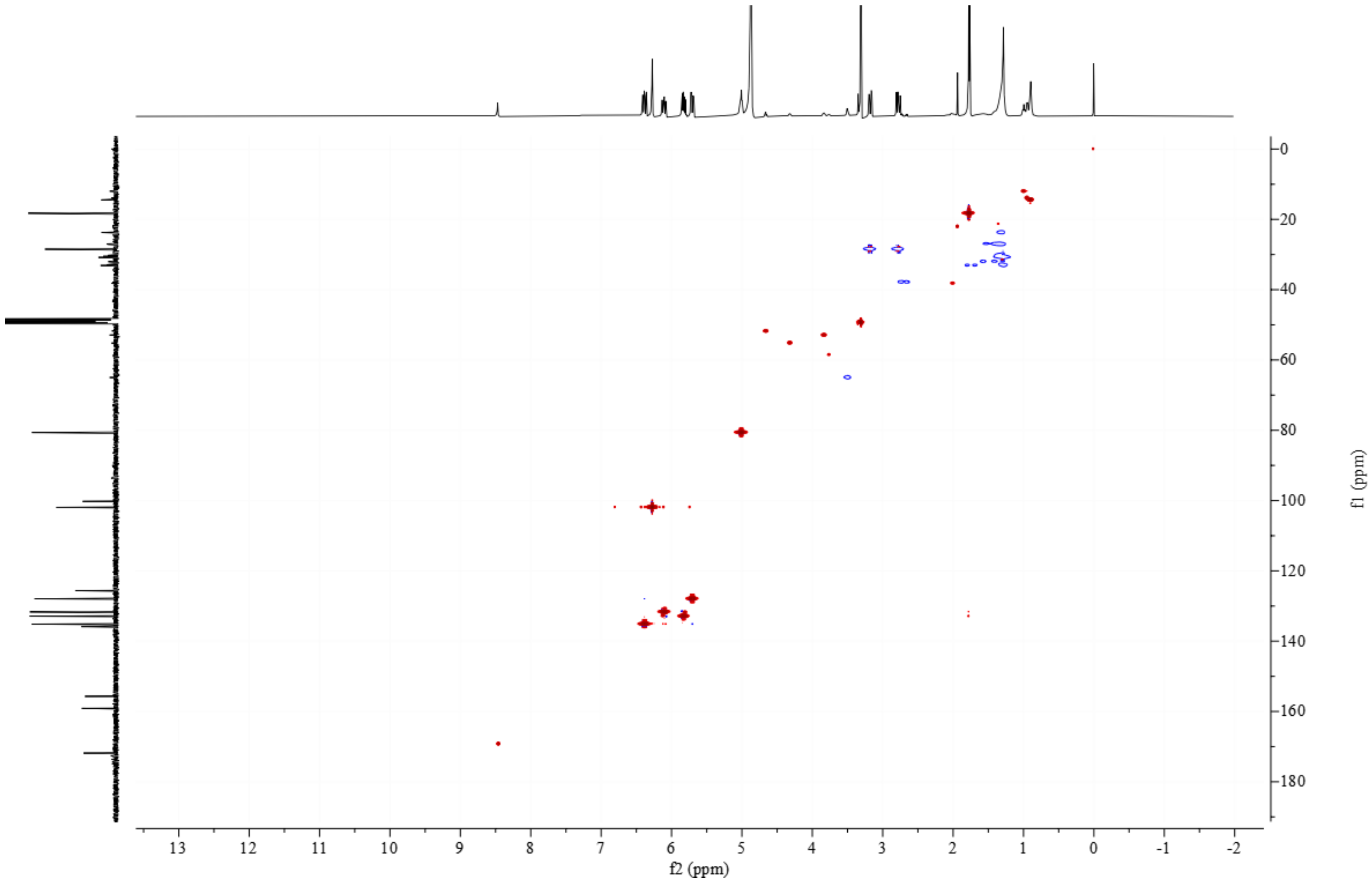

Figure S20. HSQC of compound **4** in CD_3OD

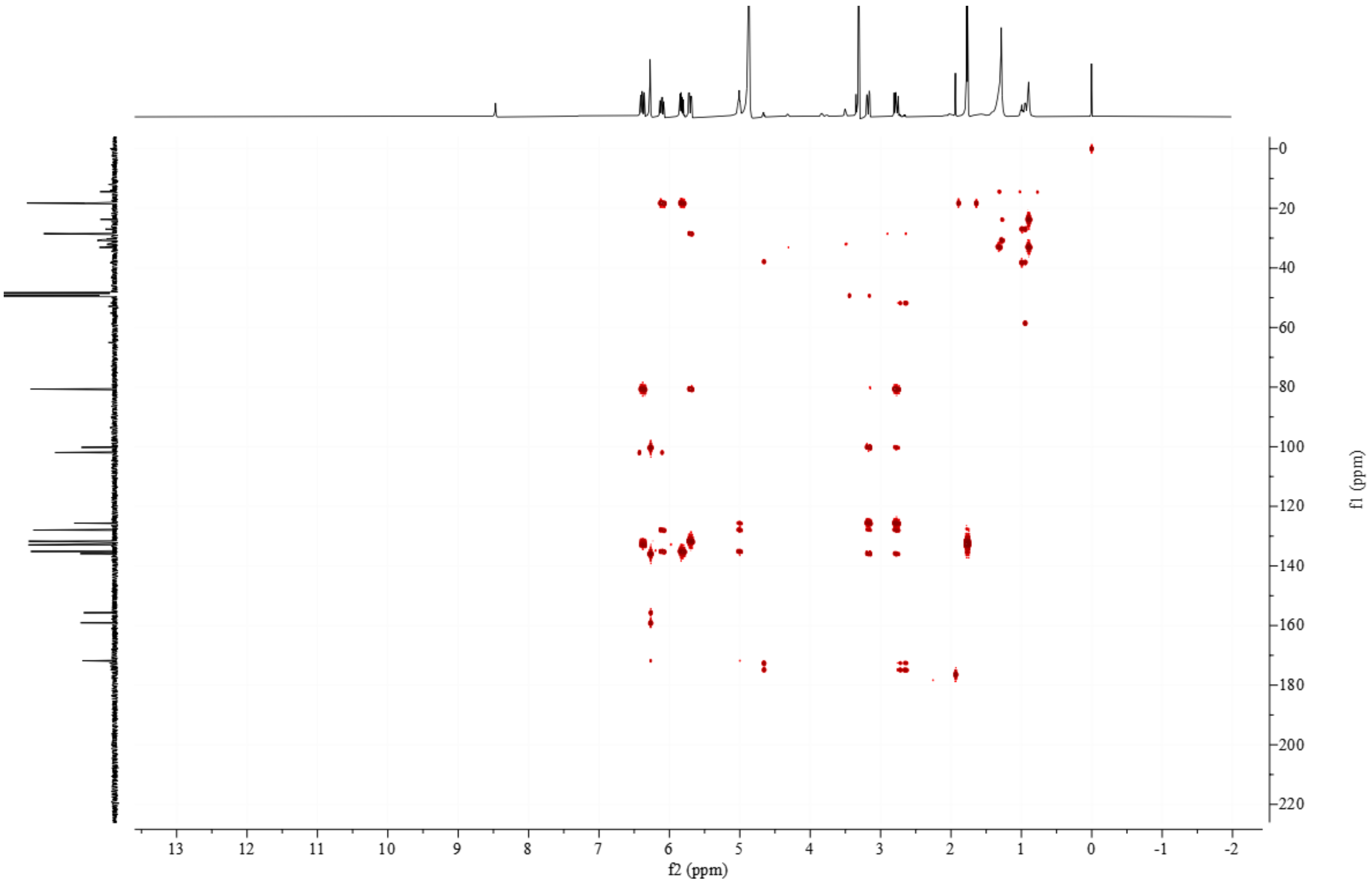

Figure S21. HMBC of compound **4** in CD_3OD

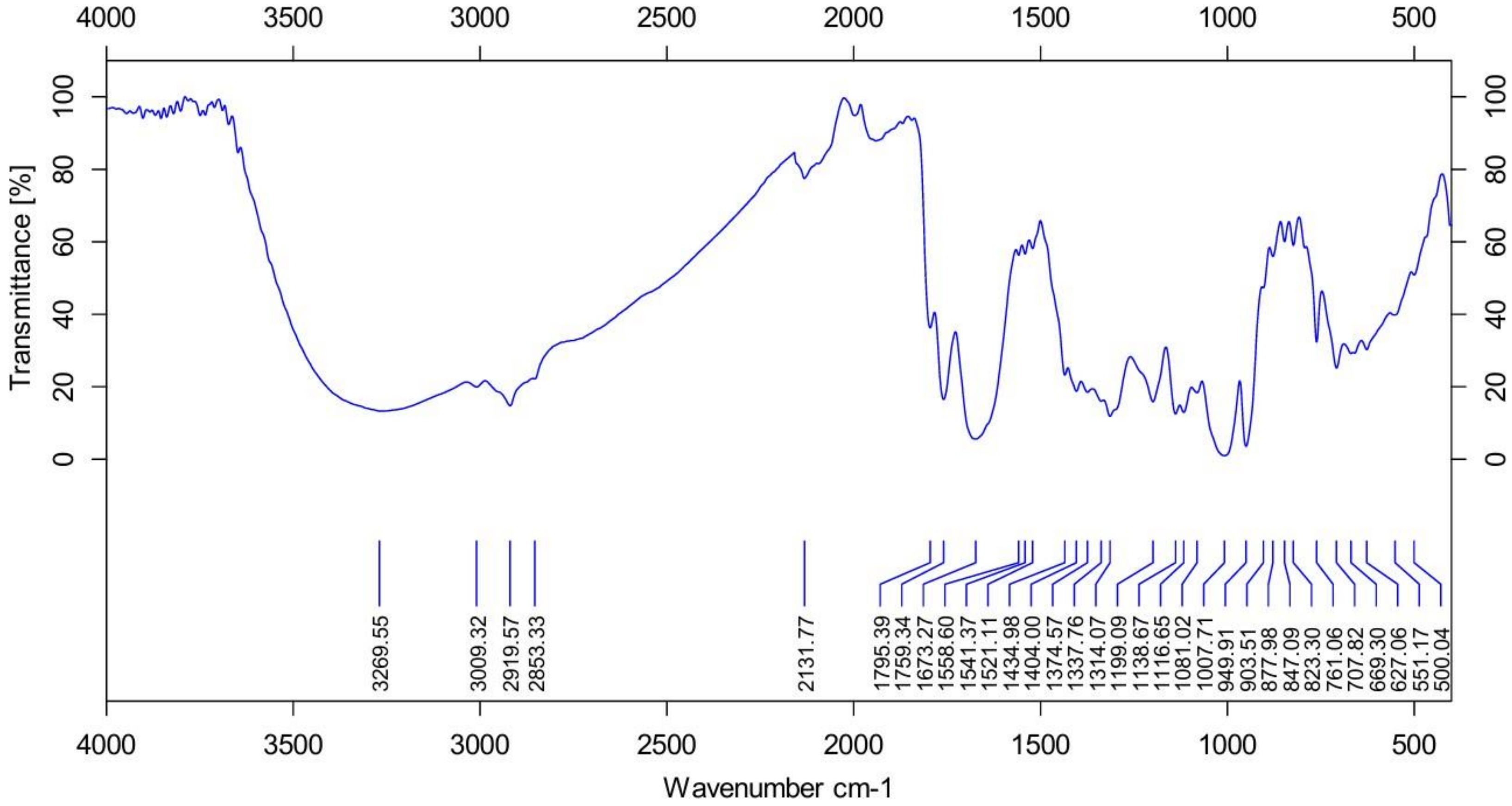

Figure S22. Infrared (IR) spectrum of compound **4**

Structure Elucidation of Known Compounds

Compound **5**: Red amorphous powder, readily soluble in methanol; $[\alpha]_D^{20}$ -22.07 (*c* 0.213, MeOH); ESI-MS *m/z* 219.10 $[M+H]^+$, molecular formula $C_{13}H_{14}O_3$; ^{1}H NMR (500 MHz, CD_3OD) δ_H 6.30 (dq, *J* = 15.2, 10.4 Hz, 1H, H-3′), 6.15 (m, 2H, H-4, 6), 6.09 (ddd, *J* = 15.0, 10.4, 1.8 Hz, 1H, H-2′), 5.75 (m, 2H, H-1′, 4′), 5.12 (q, *J* = 8.2 Hz, 1H, H-9), 3.24 (dd, *J* = 15.4, 8.9 Hz, 1H, Ha-8), 2.87 (dd, *J* = 15.4, 8.2 Hz, 1H, Hb-8), 1.75 (dd, *J* = 6.6, 1.6 Hz, 3H, H-5′); ^{13}C NMR (125 MHz, CD_3OD) δ_C 152.86 (C-5), 142.24 (C-3), 141.26 (C-2), 133.59 (C-3′), 132.01 (C-1′), 131.57 (C-2′), 130.77 (C-4′), 129.37 (C-7), 103.67 (C-6), 103.58 (C-4), 84.87 (C-9), 38.23 (C-8), 18.23 (C-5′). These data were in good agreement with those reported for asperfuran[3].

Compound **6**: Yellow solid, readily soluble in methanol; $[\alpha]_D^{20}$ +2.465 (*c* 0.284, MeOH); HR-ESI-MS *m/z* 267.1597 $[M+H]^+$ (calcd for $C_{15}H_{23}O_4$, 267.1591), molecular formula $C_{15}H_{22}O_4$; ^{1}H NMR (500 MHz, CD_3OD) δ_H 3.65 (s, 6H, H-9, 9′), 2.93 (m, *J* = 6.8 Hz, 1H, H-6), 2.11 (s, 6H, H-10, 10′), 1.76 (m, *J* = 14.1, 7.2 Hz, 1H, Ha-7), 1.39 (dt, *J* = 14.0, 7.2 Hz, 1H, Hb-7), 1.10 (d, *J* = 7.0 Hz, 3H, H-11), 0.94 (t, *J* = 7.5 Hz, 3H, H-8); ^{13}C NMR (125 MHz, CD_3OD) δ_C 211.16 (C-5), 157.34 (C-1), 155.10 (C-3, C-3′), 123.47 (C-4), 115.44 (C-2, C-2′), 62.91 (C-9, C-9′), 50.31 (C-6), 26.46 (C-7), 15.75 (C-11), 11.97 (C-8), 9.30 (C-10, C-10′). These data matched those reported for (S)-1-(4-hydroxy-2,6-dimethoxy-3,5-dimethylphenyl)-2-methylbutan-1-one.

Compound **7**: Brown amorphous powder, slightly soluble in methanol; $[\alpha]_D^{20}$ -76.19 (*c* 0.053, MeOH); HR-ESI-MS *m/z* 337.1559 $[M+H]^+$ (calcd for $C_{20}H_{21}N_2O_3$, 337.1547), molecular formula $C_{20}H_{20}N_2O_3$; ^{1}H NMR (500 MHz, DMSO-d_6) δ_H 10.84 (d, *J* = 2.3 Hz, 1H, 19-OH), 7.16 (d, *J* = 8.1 Hz, 1H, H-16), 7.06 (s, 1H, H-2), 7.02 (t, *J* = 7.5 Hz, 1H, H-15), 6.80 (d, *J* = 6.9 Hz, 1H, H-14), 4.14 (d, *J* = 11.0 Hz, 1H, H-5), 3.63 (q, *J* = 5.6 Hz, 1H, H-4), 3.05 (dd, *J* = 15.9, 12.1 Hz, 1H, Ha-12), 2.95 (dd, *J* = 15.9, 5.4 Hz, 1H, Hb-12), 2.53 (d, *J* = 5.6 Hz, 1H, H-11), 2.38 (s, 3H, H-20), 1.59 (s, 3H, H-21), 1.54 (s, 3H, H-22); ^{13}C NMR (125 MHz, DMSO-d_6) δ_C 194.75 (C-6), 184.31 (C-19), 173.47 (C-8), 133.16 (C-17), 128.73 (C-13), 125.82 (C-18), 121.95 (C-15), 121.16 (C-2), 115.43 (C-14), 108.8 (C-3), 108.7 (C-16), 105.69 (C-5), 70.84 (C-10), 62.45 (C-11), 52.56 (C-4), 35.56 (C-12), 25.95 (C-21), 25.74 (C-22), 24.32 (C-7), 20.0 (C-20). These data were consistent with those reported for α-cyclopiazonic acid[6,7].

Compound **8**: Purple-red amorphous powder, readily soluble in acetonitrile; $[\alpha]_D^{20}$ 0 (*c* 0.253, MeOH); ESI-MS *m/z* 267.06 $[M+H]^+$, molecular formula $C_{17}H_{14}O_3$; ^{1}H NMR (500 MHz, $CDCl_3$) δ_H 7.52 (br d, 2H, H-7, 11), 7.45 (m, 3H, H-8, 9, 10), 7.32 (dd, *J* = 8.0, 6.5 Hz, 2H, H-14, 18), 7.27 (d, *J* = 8.1 Hz, 1H, H-16), 7.18 (m, 2H, H-15, 17), 5.91 (s, 1H, H-5), 4.10 (d, *J* = 15.0 Hz, 1H, Ha-12), 3.80 (d, *J* = 15.1 Hz, 1H, Hb-12); ^{13}C NMR (125 MHz, $CDCl_3$) δ_C 170.81 (C-2), 158.26 (C-4), 136.11 (C-13), 130.09 (C-3),

129.35 (C-6), 129.20 (C-7, C-11), 129.15 (C-15, C-17), 129.07 (C-9), 128.95 (C-14, C-18), 128.86 (C-8, C-10), 127.39 (C-16), 96.49 (C-5), 32.55 (C-12). These data were consistent with those reported for microperfuranone[8,9].

Compound **9**: White solid, readily soluble in methanol; ESI-MS *m/z* 195.06 $[M+H]^+$, molecular formula $C_{10}H_{10}O_4$; ^{1}H NMR (700 MHz, CD_3OD) δ_H 2.16 (s, 3H, H-10), 3.89 (s, 3H, H-11), 4.48 (s, 2H, H-3), 6.93 (s, 1H, H-4); ^{13}C NMR (175 MHz, CD_3OD) δ_C 168.8 (C-1), 164.5 (C-5), 155.5 (C-7), 145.4 (C-9), 122.7 (C-8), 104.5 (C-4), 101.5 (C-6), 68.3 (C-3), 60.0 (C-11), 8.6 (C-10). These data were in agreement with those reported for nidulol[10].

Compound **10**: White solid, readily soluble in methanol; ESI-MS *m/z* 194.07 $[M+H]^+$, molecular formula $C_{10}H_{11}NO_3$; ^{1}H NMR (700 MHz, CD_3OD) δ_H 2.12 (s, 3H, H-11), 3.96 (s, 3H, H-10), 5.18 (s, 2H, H-3), 7.28 (s, 1H, H-7); ^{13}C NMR (175 MHz, CD_3OD) δ_C 171.66 (C-1), 157.6 (C-6), 156.1 (C-4), 132.6 (C-8), 123.8 (C-9), 119.8 (C-5), 104.1 (C-7), 60.2 (C-10), 40.4 (C-3), 8.6 (C-11). These data matched those reported for cichorine[11]

Method and Results of Reverse Transcription PCR (RT-PCR)

Fresh fungal mycelia were harvested, blotted dry with filter paper to remove residual medium, and immediately transferred to a pre-cooled mortar. The mycelia were thoroughly ground into a uniform fine powder with continuous addition of liquid nitrogen to maintain a frozen state throughout the grinding process, which effectively inhibited endogenous RNase activity.

Approximately 50–100 mg of the ground mycelial powder was transferred to an RNase-free centrifuge tube containing 1 mL TRIzol reagent (Invitrogen, Carlsbad, CA, USA). The mixture was vigorously vortexed to ensure complete lysis and incubated at room temperature (15–30 ℃) for 5–10 min to dissociate nucleic acid-protein complexes. Subsequently, 200 μL of chloroform was added, and the tube was shaken vigorously for 15 s until the solution became emulsified. After incubation at room temperature for 2–3 min, the mixture was centrifuged at 12,000 rpm for 15 min at 4 ℃. The upper aqueous phase (approximately 400–500 μL) was carefully transferred to a new RNase-free centrifuge tube, avoiding contamination from the interphase or organic phase.

An equal volume of isopropanol was added to the aqueous phase, and the tube was gently inverted to mix. The RNA was precipitated by incubation at room temperature for 10 min, followed by centrifugation at 12,000 rpm for 10 min at 4 ℃. The supernatant was discarded, and the white gelatinous RNA pellet was washed twice with 1 mL of pre-cooled 75% (v/v) ethanol. Each wash was followed by centrifugation at 7,500 rpm for 5 min at 4 ℃.

Residual ethanol was carefully aspirated, and the pellet was air-dried at room temperature for 5–10 min with the tube cap open (avoiding over-drying, which reduces RNA solubility). The RNA pellet was dissolved in 20–50 μL of RNase-free dH_2O by gentle pipetting. The concentration and purity of the RNA were determined using a NanoDrop 2000 spectrophotometer (Thermo Fisher Scientific, Waltham, MA, USA), with an acceptable OD_{260}/OD_{280} ratio of 1.8–2.1. RNA integrity was verified by 1% (w/v) agarose gel electrophoresis.

Genomic DNA contamination was removed prior to reverse transcription using the gDNA Eraser component of the PrimeScript™ RT Reagent Kit with gDNA Eraser (Takara, Dalian, China). The genomic DNA removal reaction mixture (total volume 10 μL) was prepared on ice as follows: 2.0 μL of 5×gDNA Eraser Buffer, 1.0 μL of gDNA Eraser, up to 2 μg of total RNA, and RNase-free dH_2O to a final volume of 10 μL. To ensure accuracy, a master mix was prepared for n+2 reactions, aliquoted into individual tubes, and then supplemented with total RNA. The reaction was incubated at room temperature for 30 min.

Reverse transcription was performed immediately after genomic DNA removal using the same kit. The reverse transcription reaction mixture (total volume 20 μL) was prepared on ice by adding 1.0 µL of PrimeScript RT Enzyme Mix I, 4.0 μL of RT Primer Mix, 4.0 μL of 5×PrimeScript Buffer 2, and 1.0 μL of RNase-free dH_2O to the 10 μL of the above genomic DNA removal reaction mixture. A master mix was prepared for n+2 reactions and aliquoted into individual tubes. The reverse transcription reaction was carried out at 37 ℃ for 15 min, followed by heat inactivation at 85 ℃ for 5 s. The synthesized cDNA was stored at -20 ℃ until further use.

Using the above cDNA as a template, the target genes were amplified by PCR. The resulting PCR products were separated by 1% (w/v) agarose gel electrophoresis in 1×TAE buffer at 120 V for 30 min and visualized under ultraviolet transillumination using a GelDoc XR+ imaging system (Bio-Rad, Hercules, CA, USA). The result is shown in Figure S23.

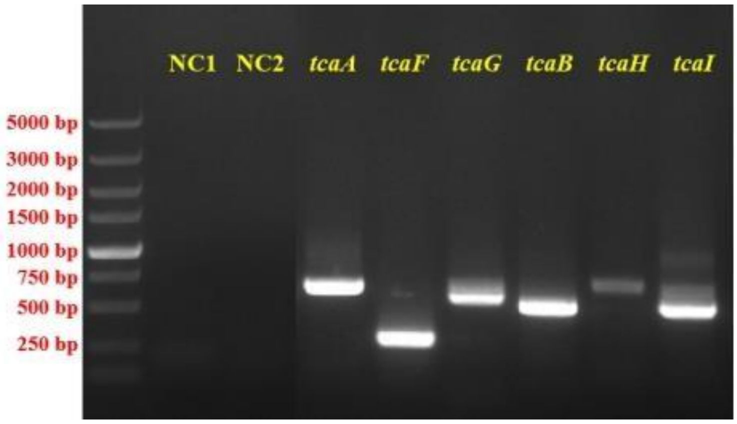

Figure S23. RT-PCR assays of partial genes in the recombinant strain AN-*tcaABCDEFGHI.* NC1 and NC2 (Negative Control): PCR amplifies non-genetic fragments (two plasmids), eliminating interference from residual DNA; Genes *tcaA,B,F-I*: PCR amplifies a partial fragment of the corresponding gene after RNA reverse transcription. The result indicated that all six exogenous genes were expressed.

Reference

[1] Bai J, Yan D, Zhang T, et al. A Cascade of Redox Reactions Generates Complexity in the Biosynthesis of the Protein Phosphatase-2 Inhibitor Rubratoxin A [J]. *Angewandte Chemie International Edition*, 2017, 56(17): 4782-4786. DOI: 10.1002/anie.201701547

[2] Bai J, Mu R, Dou M, et al. Epigenetic modification in histone deacetylase deletion strain of *Calcarisporium arbuscula* leads to diverse diterpenoids [J]. *Acta Pharmaceutica Sinica B*, 2018, 8(4): 687-697. DOI: 10.1016/j.apsb.2017.12.012

[3] Pfefferle W, Anke H, Bross M, et al. Asperfuran, a novel antifungal metabolite from *Aspergillus oryzae* [J]. The Journal of Antibiotics, 1990, 43: 648-654. DOI: 10.7164/antibiotics.43.648

[4] Orfali R, Perveen S. Secondary metabolites from the *Aspergillus* sp. in the rhizosphere soil of *Phoenix dactylifera* (Palm tree) [J]. *BMC Chemistry*, 2019, 13: 103. DOI: 10.1186/s13065-019-0624-5

[5] Barra L, Barac P, König GM, et al. Volatiles from the fungal microbiome of the marine sponge *Callyspongia* cf. *flammea* [J]. *Organic & Biomolecular Chemistry*, 2017, 15: 7411-7421. DOI: 10.1039/c7ob01837a

[6] Lin, AQ., Du, L., Fang, YC. et al. *iso-α*-Cyclopiazonic acid, a new natural product isolated from the marine-derived fungus *Aspergillus flavus* C-F-3 [J]. *Chemistry of Natural Compounds*, 2009, 45: 677-680. DOI: 10.1007/s10600-009-9433-8

[7] Christian Beyer, Jürgen Scherkenbeck, Frank Sondermann, et al. The Knight route to cyclopiazonic acid: enantioselective synthesis of a key intermediate [J]. *Tetrahedron*, 2010, 66: 7119-7123. DOI: 10.1016/j.tet.2010.06.092

[8] Fujimoto H, Asai T, Kim YP, et al. Nine constituents including six xanthone-related compounds isolated from two ascomycetes, *Gelasinospora santi-florii* and *Emericella quadrilineata*, found in a screening study focused on immunomodulatory activity [J]. *Chemical and Pharmaceutical Bulletin*, 2006, 54: 550-553. DOI: 10.1248/cpb.54.550

[9] Lü, F., Li, X., Chi, L. et al. A new acyclic peroxide from *Aspergillus nidulans* SD-531, a fungus obtained from deep-sea sediment of cold spring in the South China Sea [J]. *Journal of Oceanology and Limnology*, 2020, 38: 1225-1232. DOI: 10.1007/s00343-020-0052-3

[10] Sanchez JF, Entwistle R, Corcoran D, et al. Identification and molecular genetic analysis of the cichorine gene cluster in *Aspergillus nidulans* [J]. *Medchemcomm*, 2012, 3: 8. DOI: 10.1039/C2MD20055D

[11] Moreau A, Couture A, Deniau E, et al. First total synthesis of cichorine and zinnimidine [J]. *Organic & Biomolecular Chemistry*, 2005, 3: 2305-2309. DOI: 10.1039/b504602e
